# Heart rate variability: reference values and role for clinical profile and mortality in individuals with heart failure

**DOI:** 10.1007/s00392-023-02248-7

**Published:** 2023-07-09

**Authors:** Silav Zeid, Gregor Buch, David Velmeden, Jakob Söhne, Andreas Schulz, Alexander Schuch, Sven-Oliver Tröbs, Marc William Heidorn, Felix Müller, Konstantin Strauch, Katrin Coboeken, Karl J. Lackner, Tommaso Gori, Thomas Münzel, Jürgen H. Prochaska, Philipp S. Wild

**Affiliations:** 1 Preventive Cardiology and Preventive Medicine, Department of Cardiology, University Medical Center of the Johannes Gutenberg University Mainz, Langenbeckstr. 1, 55131 Mainz, Germany; 2 German Center for Cardiovascular Research (DZHK), Partner Site Rhine-Main, Mainz, Germany; 3 Institute of Medical Biostatistics, Epidemiology and Informatics (IMBEI), University Medical Center of the Johannes Gutenberg University Mainz, Mainz, Germany; 4 SPM Methods and Applications, Research and Development, Pharmaceuticals, BAYER AG, Wuppertal, Germany; 5 Institute of Clinical Chemistry and Laboratory Medicine, University Medical Center of the Johannes Gutenberg University Mainz, Mainz, Germany; 6 Cardiology I, Department of Cardiology, University Medical Center of the Johannes Gutenberg University Mainz, Mainz, Germany; 7 Clinical Epidemiology and Systems Medicine, Center for Thrombosis and Hemostasis (CTH), University Medical Center of the Johannes Gutenberg University Mainz, Mainz, Germany; 8 Institute of Molecular Biology (IMB), Mainz, Germany

**Keywords:** Heart failure, Autonomic dysfunction, Heart rate variability, Biomarker, Mortality

## Abstract

**Aims:**

To establish reference values and clinically relevant determinants for measures of heart rate variability (HRV) and to assess their relevance for clinical outcome prediction in individuals with heart failure.

**Methods:**

Data from the MyoVasc study (NCT04064450; *N* = 3289), a prospective cohort on chronic heart failure with a highly standardized, 5 h examination, and Holter ECG recording were investigated. HRV markers were selected using a systematic literature screen and a data-driven approach. Reference values were determined from a healthy subsample. Clinical determinants of HRV were investigated via multivariable linear regression analyses, while their relationship with mortality was investigated by multivariable Cox regression analyses.

**Results:**

Holter ECG recordings were available for analysis in 1001 study participants (mean age 64.5 ± 10.5 years; female sex 35.4%). While the most frequently reported HRV markers in literature were from time and frequency domains, the data-driven approach revealed predominantly non-linear HRV measures. Age, sex, dyslipidemia, family history of myocardial infarction or stroke, peripheral artery disease, and heart failure were strongly related to HRV in multivariable models. In a follow-up period of 6.5 years, acceleration capacity [HR_perSD_ 1.53 (95% CI 1.21/1.93), *p* = 0.0004], deceleration capacity [HR_perSD_: 0.70 (95% CI 0.55/0.88), *p* = 0.002], and time lag [HR_perSD_ 1.22 (95% CI 1.03/1.44), *p* = 0.018] were the strongest predictors of all-cause mortality in individuals with heart failure independently of cardiovascular risk factors, comorbidities, and medication.

**Conclusion:**

HRV markers are associated with the cardiovascular clinical profile and are strong and independent predictors of survival in heart failure. This underscores clinical relevance and interventional potential for individuals with heart failure.

**ClinicalTrials.gov identifier:**

NCT04064450.

**Supplementary Information:**

The online version contains supplementary material available at 10.1007/s00392-023-02248-7.

## Background

Heart failure is a complex, poly-etiologic clinical syndrome in which neurohormonal systems such as the renin–angiotensin–aldosterone system, the autonomic nervous system (ANS), and in particular the sympathetic and parasympathetic nervous systems play a pivotal role [1]. However, the involvement of the ANS in the pathophysiology of heart failure has not yet been completely understood.

Both branches of the ANS control heart rate, reflecting the sympathovagal balance of the autonomic control of the ventricles. Cardiac sympathetic nerves innervating the sinus node release catecholamine neurotransmitters, mainly epinephrine and norepinephrine, and accelerate heart rate. The parasympathetic nerves release the neurotransmitter acetylcholine, which has a local inhibitory effect in cardiomyocytes to decrease the heart rate [2]. It is known that sympathetic nervous system activity is chronically increased in heart failure, and parasympathetic nervous system activity is simultaneously inhibited, resulting in sympathetic overactivation [3]. The increase in sympathetic activity in heart failure also affects other organs, such as the kidneys and the peripheral vasculature, where it can lead to systemic vasoconstriction and augmented venous tone [4]. To measure autonomic function in cardiovascular disease, measures of heart rate variability (HRV) have been increasingly used as non-invasive markers reflecting ANS activity.

HRV is the temporal variation between heartbeats, and is assessed based on the intervals between R-waves of the QRS complex in the electrocardiogram. Several measures can be derived and are typically grouped into three domains: time domain, frequency domain, and non-linear indices. The time domain is a quantification of the temporal variability between RR intervals, the frequency domain a decomposition of the individual frequencies that make up the electrical pulses emitted by the sinus node, and non-linear indices capture the stochasticity of the overall heart rate variability. The direct translation from ANS activity to HRV measures has not been fully elucidated [5].

Investigations using HRV measures by ambulatory Holter ECG are scarce or outdated, even though 24 h ECG recordings more accurately reflect normal daily physical activity than standardized short-term HRV measurements [6]. Moreover, reference values for HRV measures derived from Holter ECG have not been established yet. Several studies have shown that a large reduction in HRV is associated with cardiac and all-cause death when assessed with short-time measurements of HRV [7–9]. However, the prognostic value of long-term HRV markers for survival in patients with heart failure has not been comprehensively investigated, which represents an important gap in the evidence for the clinical relevance of HRV in heart failure.

The aims of this study were (i) to identify clinically relevant measures of HRV, (ii) to determine normal values of HRV measures, (iii) to identify clinical determinants of HRV measures, and (iv) to assess the clinical relevance of HRV measures for the clinical status and all-cause and cardiac mortality in individuals with heart failure.

## Methods

### Study design

For this study, data from the MyoVasc study (ClinicalTrials.gov Identifier: NCT04064450), a prospective, observational cohort study of individuals with heart failure (*N* = 3289), were analyzed. The local data protection officer and the responsible ethics committee approved the study protocol [reference number 837.319.12 (8420-F)] prior to study initiation. All study participants provided written informed consent prior to study enrollment. The Declaration of Helsinki [10] and the recommendations of good clinical practice and good epidemiological practice were followed in all study procedures. Study participants were recruited from hospitals, practices and by random sampling from registry offices.

All participants in the MyoVasc study were classified into stages of heart failure according to the current Universal Definition of Heart Failure as proposed by the writing committee of the Heart Failure Society of America, Heart Failure Association of the European Society of Cardiology, and the Japanese Heart Failure Society [11]. Echocardiography was performed in the MyoVasc study center, as part of the examination, to assess the cardiac status. A detailed description of the design and rationale for the MyoVasc study, including baseline characteristics, has been published recently [12].

### Study participant examination

Participants underwent an extensive 5 h array of medico-technical measurements, including the assessment of cardiac structure and function by transthoracic cardiac echocardiography. Venous blood was drawn to measure routine blood markers relevant to traditional cardiovascular risk factors (CVRFs) and disease, and the heart failure syndrome in particular (see Supplemental Text 1 for a detailed description). Presence of CVRFs (arterial hypertension, diabetes mellitus, smoking, obesity, dyslipidemia, family history of ischemic stroke or myocardial infarction) and comorbidities (myocardial infarction, stroke, coronary artery disease, peripheral artery disease defined as physicians’ diagnose or ankle-brachial index [ABI] < 0.9, cancer, chronic kidney disease defined as physicians’ diagnose or estimated glomerular filtration rate [eGFR] < 60 ml/min/1.73 m^2^ [CKD], venous thromboembolism [VTE] and chronic obstructive pulmonary or airway disease) was evaluated during an extensive computer-assisted personal interview, and assessed by laboratory markers and clinical investigations where applicable. Medication intake was classified according to the Anatomical Therapeutic Chemical Classification System (ATC). With the baseline examination, participants received a multi-channel (3-lead) digital 24 h Holter ECG recorder (resolution storage: 1024 Hz/12bit; Cardiomem^®^ CM 4000, GETEMED, Teltow, Germany) in ambulatory setting. Study participants were instructed regarding the correct handling of the devices during day and nighttime and asked to keep a log of their physical activity. The SQUASH (Short Questionnaire to Assess Health enhancing physical activity) instrument was used to assess the habitual physical activity level.

### Assessment of heart rate variability

Holter ECG data were transferred from the device and imported into Holter ECG analysis software (CardioDay^®^ 2.4.3.16, GETEMED) to retrieve RR intervals. Subsequently, RR intervals were analyzed and filtered for artifacts, prior to the conversion into 70 secondary variables from the time domain, frequency domain, and non-linear indices of HRV using the ‘RHRV’ R package [13]. A detailed description and overview of all HRV parameters computed for the MyoVasc study can be found in the extended methods section in Supplemental Text 1 and Supplemental Table 1, respectively.

### Marker selection, data handling, and statistical analysis

For this analysis, only participants with 24 h Holter ECG data were included. Participants with pacemaker stimulation and those in whom atrial fibrillation was present during at least half of the Holter ECG recording time were excluded, as well as participants who underwent a heart transplantation. HRV markers of interest were selected using (1) a systematic literature screen on markers relevant for cardiovascular disease and (2) a random survival forest model of markers for predicting cardiac death. A literature screen on PubMed was conducted for every HRV parameter using a search query including the respective HRV parameter, the term ‘HRV’, and the term ‘cardiovascular’ (detailed search queries provided in Supplemental Table 2). The search results were ranked by frequency of search hits, in descending order. The top ten ranked HRV markers were considered to be important according to the HRV-related scientific knowledge. A random survival forest model was fitted to rank HRV markers in relation to cardiac death adjusted for age and sex. Minimum depth was used as variable importance metric, where a lower value corresponds to greater importance for the prediction. The top ten ranked HRV markers were again considered clinically important. The combined result from the literature screen and machine learning approach was a set of 20 relevant HRV markers.

The distribution of these 20 HRV markers was determined in a subgroup of study participants classified as healthy or at risk of heart failure (heart failure stage A), and in individuals with heart failure (i.e., heart failure stage B, C, or D). The first group served as reference group after exclusion of participants with diabetes mellitus diagnosed ≥ 10 years ago who were not receiving dietary treatment, as well as participants with degenerative or structural neurological disorders. Values corresponding to the 5th and 95th percentiles were taken as bounds of the reference range.

The age of the study participants was reported as mean [standard deviation, (SD)], and discrete variables were described by relative and absolute frequencies, stratified by the reference group and individuals with heart failure. Multivariable linear regression models were used to exploratively investigate the relationship between the clinical profile (i.e., cardiovascular risk factors and comorbidities), and the selected HRV markers in individuals with heart failure. Linear regression models were adjusted for age, sex, cardiovascular risk factors, comorbidities, and medication intake, i.e., antidiabetic agents, antithrombotic agents, cardiac therapy, diuretic agents, beta-receptor blocking agents, selective beta-blocking agents, calcium channel blocker, agents acting on the renin–angiotensin system, ACE inhibitors, angiotensin-II-receptor blockers, lipid modifying agents, and antidepressants. Dependent variables (i.e., HRV markers) were standardized for comparisons, i.e., divided by their standard deviations. Finally, all-cause mortality over 8 years of follow-up and cardiac death over 6 years of follow-up in individuals with heart failure were shown for each HRV marker stratified by tertiles, and by their values being within or outside the reference range. Multivariable Cox proportional hazard regression models, adjusted for age, sex, and additionally for cardiovascular risk factors, comorbidities, and medication intake, were computed to examine the independent contribution of standardized HRV markers to all-cause mortality and cardiac death. Since this was an exploratory investigation, *p* values were interpreted as continuous measures of statistical evidence for an association. A two-sided *p* value < 0.05 was considered a nominally significant association. All analyses were performed in R, version 4.0.3 (R Foundation for Statistical Computing, Vienna, Austria).

## Results

### Baseline characteristics of study participants

The analysis sample comprised 1001 study participants [mean age in years (SD): 64.5 (10.5); female sex: 35.4%; see Supplemental Fig. 1 for the study flow chart). The mean wearing time of the ambulatory Holter ECG device was 22.49 (± 4.12) h. Baseline characteristics for study participants with heart failure and the reference group are presented in Table 1. Characteristics of functional and structural cardiac status markers are reported in Supplemental Table 3. The median concentration of NT-proBNP in participants with heart failure was 204.1 pg/ml [interquartile range (IQR) 96.0/491.2 pg/ml]. The reference group was drawn from the analysis sample and contained 133 individuals after excluding study participants classified as heart failure stage B (*n* = 301), C or D (*n* = 554), participants diagnosed with diabetes mellitus ≥ 10 years ago not receiving dietary treatment (*n* = 10), and participants with degenerative or structural neurological disorders (*n* = 3). All traditional cardiovascular risk factors were more prevalent among individuals with heart failure than the reference group. This was most pronounced for arterial hypertension (heart failure: 77.3 vs. 53.4% in the reference group), dyslipidemia (78.9 vs. 48.1%), and obesity (33.6 vs. 22.6%). The prevalence of cancer, COPD, and VTE were approximately equal between individuals with heart failure and the reference group. In contrast, atherosclerotic cardiovascular disease (i.e., coronary artery disease, history of myocardial infarction, stroke or TIA, and peripheral artery disease) was more prevalent in heart failure as compared to the reference group.

**Table 1:** Sample characteristics of the analysis sample with heart failure and the reference group

	Heart failure (*n* = 855)	Reference group (*n* = 133)
Demographics		
Female sex, [%] (*n*)	33.9 (290)	46.6 (62)
Age, [years]	65.6 (10.1)	57.8 (10.3)
Heart failure stages		
Heart failure stage 0/A, [%] (*n*)	0 (0)	100 (133)
Heart failure stage B, [%] (*n*)	35.2 (301)	0 (0)
Heart failure stage C/D, [%] (*n*)	64.8 (554)	0 (0)
Cardiovascular risk factors		
Active smoking, [%] (*n*)	11.7 (100)	7.5 (10)
Arterial hypertension, [%] (*n*)	77.3 (661)	53.4 (71)
Diabetes mellitus, [%] (*n*)	23.6 (202)	6.8 (9)
Dyslipidemia, [%] (*n*)	78.9 (675)	48.1 (64)
Family history of MI or stroke, [%] (*n*)	25.6 (218)	24.1 (32)
Obesity, [%] (*n*)	33.6 (287)	22.6 (30)
Comorbidities		
Atrial fibrillation, [%] (*n*)	24.0 (205)	8.3 (11)
Chronic kidney disease, [%] (*n*)	25.4 (216)	12.0 (16)
Chronic obstructive pulmonary disease, [%] (*n*)	13.7 (117)	13.5 (18)
Coronary artery disease, [%] (*n*)	48.8 (417)	15.8 (21)
History of cancer, [%] (*n*)	16.7 (143)	16.5 (22)
History of myocardial infarction, [%] (*n*)	34.0 (291)	0 (0)
History of stroke, [%] (*n*)	8.8 (75)	3.8 (5)
History of transient ischemic attack, [%] (*n*)	6.4 (55)	1.5 (2)
History of venous thromboembolism, [%] (*n*)	9.8 (84)	5.3 (7)
Pacemaker, [%] (*n*)	2.5 (21)	0.8 (1)
Peripheral artery disease, [%] (*n*)	7.7 (66)	0.8 (1)
Medication		
Agents acting on the RAS (C09), [%] (*n*)	74.3 (635)	38.3 (51)
Antidepressants (N06A), [%] (*n*)	9.2 (79)	8.3 (11)
Antidiabetic agents (A10), [%] (*n*)	17.1 (146)	6 (8)
Antithrombotic agents (B01A), [%] (*n*)	73.1 (625)	30.1 (40)
Beta blockers (C07), [%] (*n*)	66.8 (571)	18.0 (24)
Calcium channel blocker (C08), [%] (*n*)	19.9 (170)	10.5 (14)
Digitalis glycosides, anti-arrhythmics, and vasodilators (C01), [%] (*n*)	21.5 (184)	5.3 (7)
Diuretic agents (C03), [%] (*n*)	35.2 (301)	8.3 (11)
Lipid modifying agents (C10), [%] (*n*)	55.7 (476)	26.3 (35)

Heart failure defined according to the Universal Definition of Heart Failure as stated in the report of the Heart Failure Society of America, Heart Failure Association of the European Society of Cardiology, Japanese Heart Failure Society and Writing Committee of the Universal Definition of Heart Failure, stage B, C or D; The reference group is defined as healthy or at risk of heart failure (HF stage A), excluding individuals with diabetes mellitus diagnosed ≥ 10 years ago who were not receiving dietary treatment, as well as participants with degenerative or structural neurological disorders. Categorical variables are presented in relative and absolute frequencies. Age is reported as mean and standard deviation. Data were available in 97.3% of the analysis sample. Anatomical Therapeutic Chemical Classification System (ATC) codes for each type of medication are displayed in brackets

*MI* myocardial infarction, *RAS* renin–angiotensin system

### Selection of clinically relevant HRV parameters

**Fig. 1:**
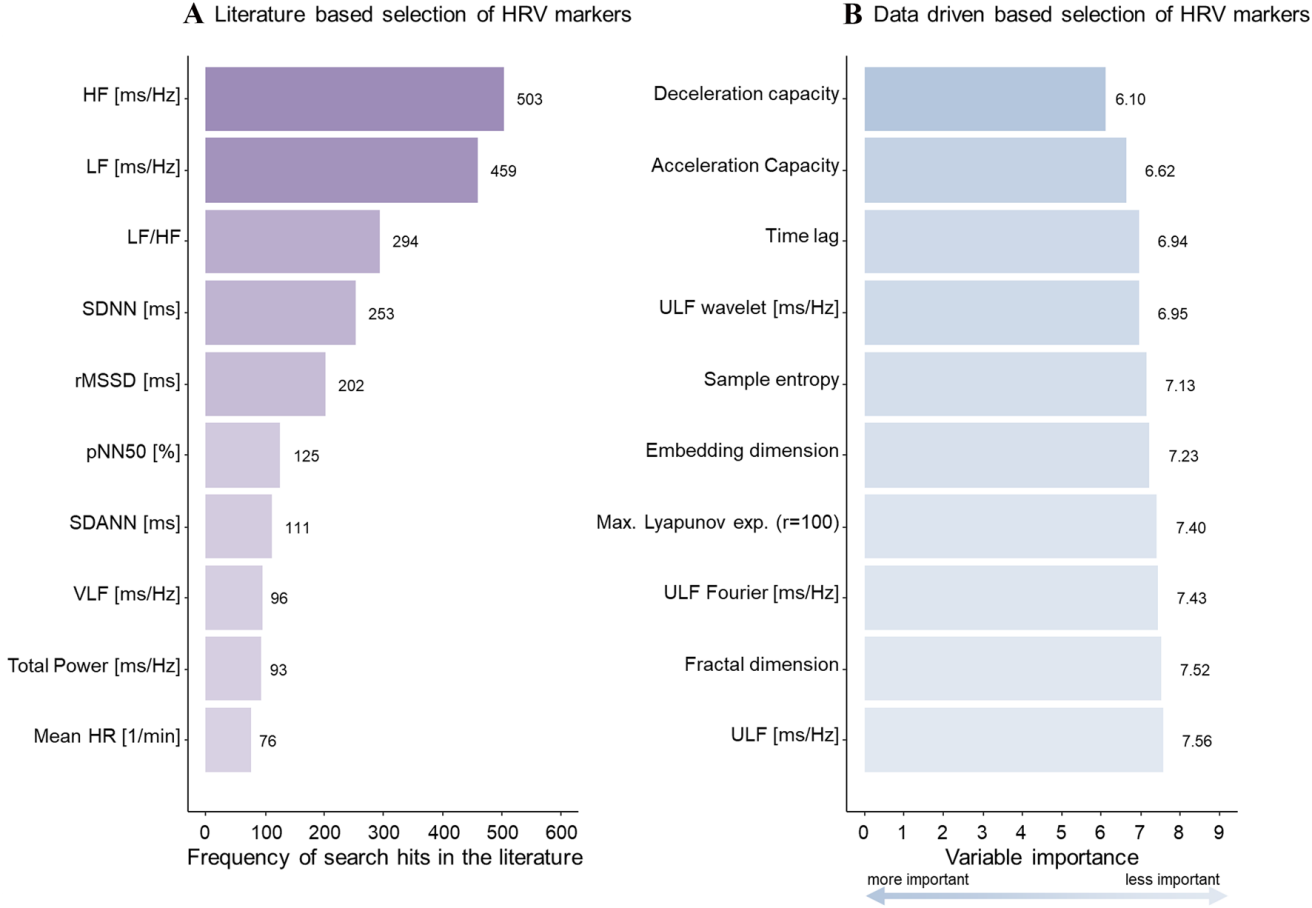
Selection of heart rate variability parameters based on a literature search (**A**) and on a data-driven approach (**B**). Left (**A**), the top ten HRV markers retrieved by a systematic literature search from the PubMed database. Right (**B**), the top 10 HRV markers identified with a random survival forest model with cardiac death as outcome and 62 HRV markers as predictors, adjusted for age and sex, ranked according to the variable importance metric minimal depth, with lower values indicating higher importance. The markers retrieved via the systematic literature search all belong to the time and frequency domains, while the top ten ranked markers from the machine learning model belong mostly to non-linear indices of HRV. The full model is displayed in Supplemental Table 4. HRV, heart rate variability; HF, high frequency; LF, low frequency; SDNN, standard deviation of the NN (normal to normal) intervals in milliseconds; rMSSD, root mean square of successive differences between normal heartbeats; pNN50%, percentage of neighboring NN intervals that differ from each other by more than 50 ms; SDANN, standard deviation of the 5 min average NN intervals; VLF, very low frequency; HR, heart rate; ULF, ultra-low frequency; Max., maximal; r, radius

**Fig. 2:**
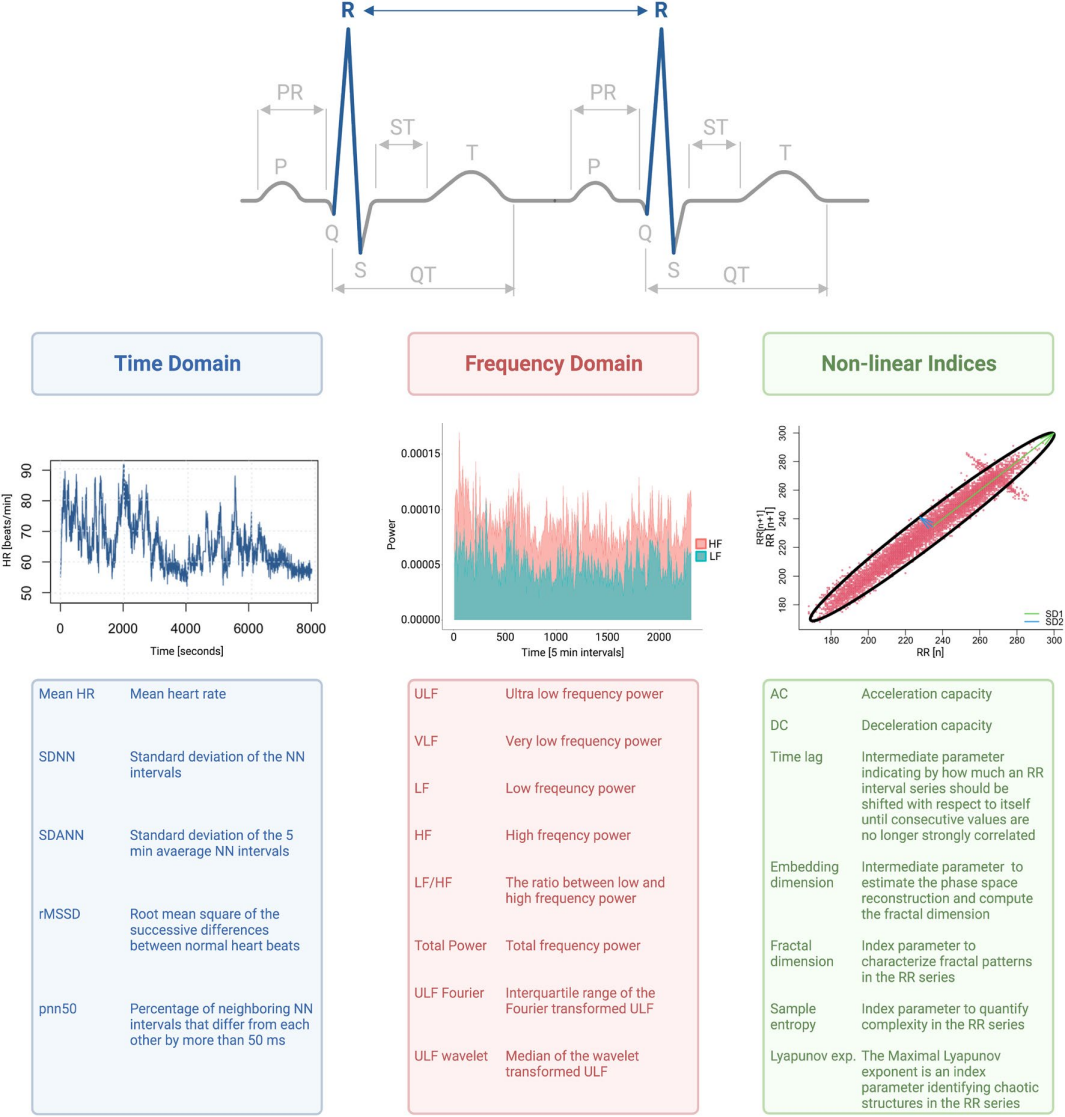
Graphical representation of the heart rate variability domains, and an overview of the different HRV abbreviations and their meanings. Graphical overview on the three different domains of HRV and an overview of the different HRV abbreviations and their meanings for the identified HRV parameters

The selection of relevant HRV markers based on systematic literature search and random survival forest model predicting cardiac death are shown in Fig. 1. The top ten HRV markers reported in literature all belonged to the time or frequency domain. The search hits for all HRV parameters are displayed in Supplemental Table 2. The top ten HRV markers from the random survival forest model, ranked by the variable importance metric minimal depth, were primarily non-linear indices and from the frequency domain. Supplemental Table 4 lists the variable importance ranking for all HRV markers. Figure 2 shows the three different HRV domains graphically, and provides an overview of the different HRV abbreviations and their meanings.

### Reference values for time, frequency, and non-linear HRV parameters

Median, IQR, and reference values for each of the HRV parameters are displayed by domain in Table 2, together with an overview of the frequencies of individuals with heart failure outside the reference range. Distributions of HRV parameters are shown in Supplemental Fig. 2.

**Table 2:** Distribution of heart rate variability and reference values in the reference sample and the sample with heart failure

	Distribution of HRV in the reference sample w/o heart failure (*N* = 133)			Distribution of HRV in the heart failure sample (*N* = 855)			Individuals with heart failure outside of the reference range		
	Median (Q1, Q3)	5th pct	95th pct	Median (Q1, Q3)	5th pct	95th pct	Outside reference range, [% (*n*)]	< 5th pct, [% (*n*)]	> 95th pct, [% (*n*)]
Time domain									
Mean HR [1/min]	77.9 (71.7/83.2)	58.7	90.4	72.2 (65.0/79.2)	56.6	91.8	14.4 (123)	7.5 (64)	6.9 (59)
SDNN [ms]	136 (113/160)	80	211	125 (101/155)	69.1	207	14.4 (123)	10.2 (87)	4.2 (36)
SDANN [ms]	121 (97.5/143)	69.7	189	110 (86.8/136)	57.8	182	15.1 (129)	11.1 (95)	4.0 (34)
rMSSD [ms]	28.2 (22.8/37.9)	14.8	71.3	32.4 (22.6/55.2)	14.0	135	22.2 (190)	6.3 (54)	15.9 (136)
pNN50 [%]	4.31 (2.57/9.70)	0.675	25.0	5.43 (2.15/13.2)	0.559	42.4	17.2 (147)	6.0 (51)	11.2 (96)
Frequency domain									
ULF [ms/Hz]	6090 (4169/8191)	2260	13,498	5134 (3303/7613)	1558	13,190	16.3 (139)	11.8 (101)	4.5 (38)
VLF [ms/Hz]	149 (102/241)	60.2	427	119 (70.4/209)	27.7	511	26.9 (230)	19.6 (168)	7.3 (62)
LF [ms/Hz]	249 (160/457)	72.1	817	190 (99.8/437)	37.7	1,551	28.1 (240)	16.0 (137)	12.1 (103)
HF [ms/Hz]	94.6 (54.6/150)	22.8	386	95.4 (46.1/253)	18.2	1,341	24.2 (207)	7.5 (64)	16.7 (81)
LF/HF	2.69 (1.90/4.27)	1.06	6.86	1.87 (1.22/2.71)	0.721	5.13	19.9 (170)	18.1 (155)	1.8 (15)
Total power [ms/Hz]	6646 (4522/9200)	2514	14,481	5817 (3785/8603)	1828	14,637	17.1 (146)	10.8 (92)	6.3 (54)
ULF Fourier [ms/Hz]	618 (398/902)	252	1603	496 (309/795)	122	1563	22.0 (188)	17.2 (147)	4.8 (41)
ULF wavelet [ms/Hz]	9904 (6114/15561)	3292	26,249	7617 (4504/13,053)	1880	26,506	19.2 (164)	14.2 (121)	5.0 (43)
Non-linear indices									
Acceleration capacity	− 6.21 (− 7.64/− 4.98)	− 9.94	− 3.60	− 4.87 (− 6.39/− 3.57)	− 8.74	− 2.17	28 (239)	2.1 (18)	25.9 (221)
Deceleration capacity	6.33 (5.02/7.74)	3.44	10.0	4.93 (3.64/6.36)	2.19	8.74	23.0 (197)	21.6 (185)	1.4 (12)
Time lag	6.0 (4.0/10.0)	2	27	8.0 (4.0/17.0)	1.00	60	33.7 (288)	16.5 (141)	17.2 (147)
Embedding dimension	9.0 (8.76/10.0)	8	12	9.31 (9.0/10.0)	7.00	12.0	28.1 (240)	20.6 (176)	7.5 (64)
Fractal dimension	2.97 (2.55/3.63)	2.09	4.72	3.12 (2.58/3.67)	1.94	4.70	13.8 (118)	9.1 (78)	4.7 (40)
Sample entropy	0.363 (0.311/0.407)	0.253	0.481	0.363 (0.311/0.413)	0.243	0.502	13.6 (116)	6.1 (52)	7.5 (64)
Max. Lyapunov exp._r=100_	0.027 (− 0.021/0.069)	− 0.13	0.155	0.022 (− 0.012/0.057)	− 0.108	0.161	8.8 (75)	3.4 (29)	5.4 (46)

*Q1* first quartile, *Q3* third quartile, *pct* percentile, *HR* heart rate, *SDNN* standard deviation of the NN intervals, *SDANN* standard deviation of the 5 min average NN intervals, *rMSSD* root mean square of the successive differences between normal heart beats, *pNN50* percentage of neighboring NN intervals that differ from each other by more than 50 ms, *ULF* ultra-low frequency, *VLF* very low frequency, *LF* low frequency, *HF* high frequency, *LF/HF* the ratio between low and high frequency, *ULF Fourier* interquartile range of the ULF short time Fourier transform, *ULF wavelet* median of the ULF wavelet transform, *Max*. maximal*, exp*. Exponent, *r* radius

### Clinical profile and heart rate variability

**Fig. 3:**
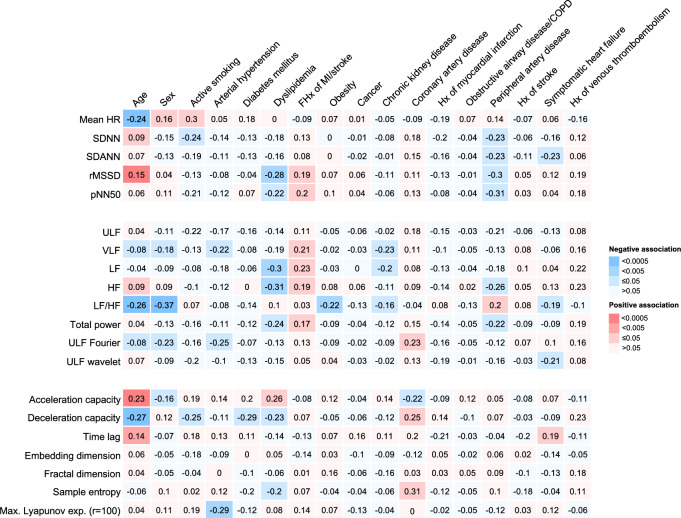
Relationships between cardiovascular risk factors, comorbidities, and heart rate variability in individuals with heart failure *N* = 855. Each cell shows the coefficient estimate and confidence interval for a cardiovascular risk factor or comorbidity from a separate linear regression model with a HRV marker (standardized) as the dependent variable, adjusted for age, sex, traditional cardiovascular risk factors, comorbidities, and medication intake in the heart failure analysis sample. The table is color coded according to the *p* values. HR, heart rate; SDNN, standard deviation of the NN intervals; SDANN, standard deviation of the 5 min average NN intervals; rMSSD, root mean square of the successive differences between normal heart beats; pNN50, percentage of neighboring NN intervals that differ from each other by more than 50 ms; ULF, ultra-low frequency; VLF, very low frequency; LF, low frequency; HF, high frequency; LF/HF, the ratio between low and high frequency; ULF Fourier, interquartile range of the ULF short time Fourier transform; ULF wavelet, median of the ULF wavelet transform; Max., maximal; exp., exponent; r, radius

The relationship between the clinical profile and HRV measures independently of age, sex, CVRFs, comorbidities, and medication in the heart failure analysis sample is shown in Fig. 3, where standardized beta-coefficients from separate linear regression models are displayed, stratified by HRV domain. Supplemental Fig. 4 and 5 include the 95% confidence intervals (95% CI), and adjustments for age and sex only.

Age and dyslipidemia were strongly associated with HRV across all three domains, while sex, a positive family history of myocardial infarction or ischemic stroke, and peripheral artery disease were only strongly associated with time and frequency domain, but not with non-linear parameters. Arterial hypertension, coronary artery disease, and heart failure were related with frequency domain parameters and non-linear indices, and smoking with time domain parameters and non-linear indices. Diabetes mellitus demonstrated a strong relationship with non-linear indices of HRV, and obesity and CKD with frequency domain parameters. Myocardial infarction, stroke, cancer, venous thromboembolism, and COPD had no clinically relevant relationships with HRV.

### HRV is prognostic for risk of death and cardiac death

The median follow-up time for assessing all-cause death and cardiac death was 6.5 years (IQR 5.1/7.5) and 6.0 years (IQR 5.1/6.0), respectively. In total, 118 participants died during the follow-up period, of whom 34 were reported to have died of a cardiac cause.

**Fig. 4:**
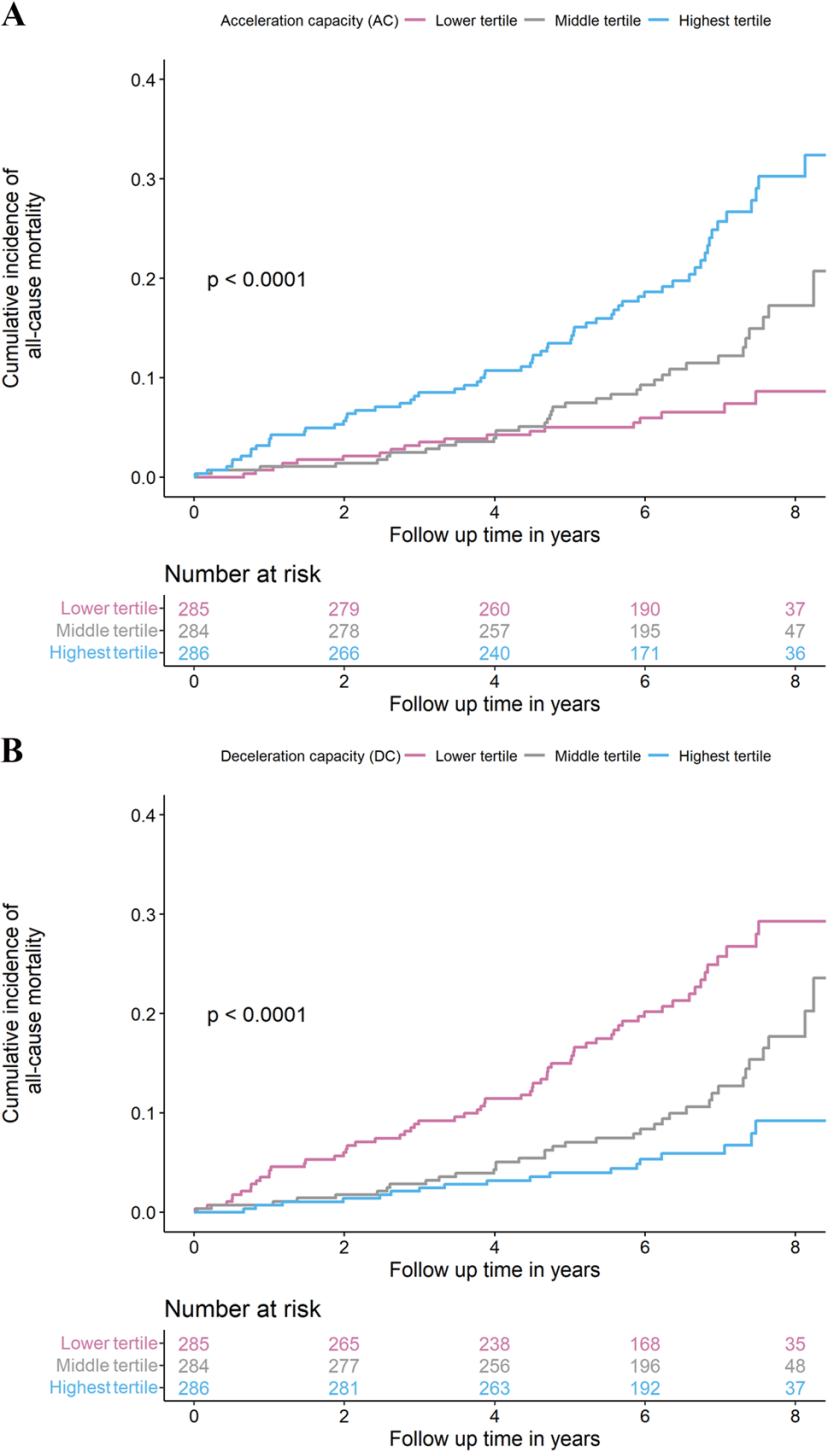
Cumulative incidence curves for all-cause mortality according to tertiles of acceleration capacity and deceleration capacity in heart failure, stage B to D. The cumulative incidences of all-cause mortality in individuals with heart failure over 8 years of follow-up stratified by tertiles are shown for the top two heart rate variability markers, acceleration capacity (panel **A**) and deceleration capacity (panel **B**), from the random survival forest model predicting cardiac death. The tertiles were for acceleration capacity: lowest tertile: ≤  − 5.86, middle tertile: >  − 5.86 and ≤  − 3.97, and highest tertile: >  − 3.97; and for deceleration capacity: lowest tertile: ≤ 4.03, middle tertile: > 4.03 and ≤ 5.71, and highest tertile: > 5.71

The cumulative incidence curves for all-cause mortality in the heart failure sample for the two highest ranked HRV predictors from the random survival forest model, acceleration capacity (AC) and deceleration capacity (DC), respectively, surrogates of sympathetic and parasympathetic nervous system activity, are shown in Fig. 4. Participants with values in the highest tertile for AC and the lowest tertile for DC had a cumulative 8 years mortality of > 30 percent, whereas those in the lowest tertile for AC and highest tertile for DC had < 10 percent in this time period. The cumulative incidence curves for all-cause death and cardiac death in the analysis sample, stratified by tertiles and values inside vs. outside the reference ranges, are shown in Supplemental Fig. 5 and 6, respectively.

**Fig. 5:**
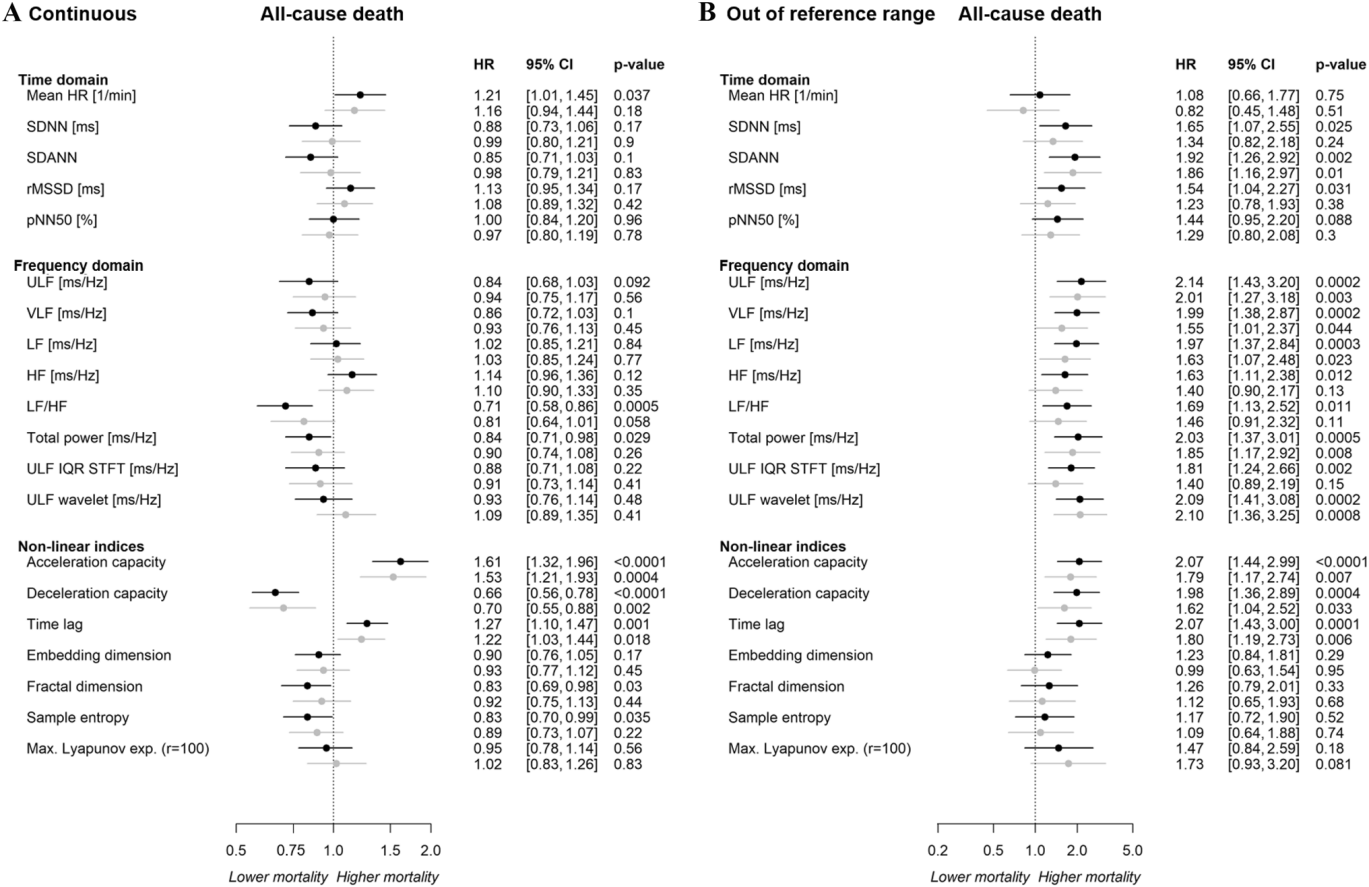
Relationship of HRV with all-cause death (**A**) as continuous trait and (**B**) for values outside of the reference range. Results of separate Cox regression models for each HRV parameter with adjustment for age and sex (black) and additional adjustment for traditional cardiovascular risk factors, comorbidities, and medication intake (grey) in the heart failure sample (*N* = 855). HRV is used as predictor and all-cause death as outcome with HRV as continuous trait (**A**) and for values outside vs. inside the reference range (**B**). HR, heart rate; SDNN, standard deviation of the NN intervals; SDANN, standard deviation of the 5 min average NN intervals; rMSSD, root mean square of the successive differences between normal heart beats; pNN50, percentage of neighboring NN intervals that differ from each other by more than 50 ms; ULF, ultra-low frequency; VLF, very low frequency; LF, low frequency; HF, high frequency; LF/HF, the ratio between low and high frequency; ULF IQR STFT, interquartile range of the ULF short time Fourier transform; ULF wavelet, median of the ULF wavelet transform; Max., maximal; exp., exponent; r, radius

Multivariable Cox regression models were used to evaluate whether HRV markers were prognostic of all-cause death in the heart failure sample independent of age, sex, CVRFs, comorbidities, and medication intake (Fig. 5A). From the time and frequency domain, the mean heart rate [HR 1.21 (95% CI 1.01–1.45), *p* = 0.04], LF/HF [HR 0.71 (95% CI 0.58–0.86), *p* = 0.0005], and total power [HR 0.84 [95% CI 0.71–0.98), *p* = 0.03] were relevantly prognostic for all-cause mortality in the age- and sex-adjusted models. However, after additional adjustment for CVRFs, comorbidities and medication intake, no association remained for time and frequency domain parameters.

Acceleration capacity [HR 1.61 (95% CI 1.32–1.96), *p* < 0.0001], DC [HR 0.66 [95% CI 0.56–0.78), *p* < 0.0001], and time lag [HR 1.27 (95% CI 1.10–1.47), *p* = 0.001], were predictive non-linear indices for all-cause mortality in both the age- and sex-adjusted models as well as the models additionally adjusted for CVRFs, comorbidities, and medication intake. The remaining markers were only weakly related to death. Further adjustments for left ventricular ejection fraction (LVEF) or physical activity did not relevantly change the relationship between HRV and outcome (Supplemental Figs. 7 and 8).

Multivariable Cox regression models demonstrated that the established reference ranges of the majority of HRV markers were independently prognostic for mortality (Fig. 5B) and cardiac death in individuals with heart failure (Supplemental Fig. 9).

With regard to the HF phenotypes, only non-linear indices of HRV are of prognostic relevance. Supplemental Fig. 10 shows that only AC, DC, and fractal dimension are prognostically relevant for all-cause death in individuals with HFpEF and only the Lyapunov exponent is prognostic in individuals with HFrEF.

## Discussion

This study introduced possible reference ranges and clinical determinants for markers of HRV in a comprehensive approach. The deep phenotyping performed in the MyoVasc study allowed for consideration of a large number of potential determinants and confounders. Markers of HRV from all three domains were prognostic of death in individuals with heart failure. Particularly values outside of the proposed reference range were strongly prognostic for clinical outcome, suggesting their relevance for clinical decision-making. The prognostic relevance of HRV out of reference range was independent of traditional CVRFs, comorbidities, and medication intake, suggesting a role for risk stratification and potential intervention strategies.

Cardiovascular risk factors associated with the development and progression of heart failure, such as arterial hypertension [14, 15], type 2 diabetes mellitus and pre-diabetes [16, 17], metabolic syndrome [18, 19], and smoking [20] have all been associated with autonomic imbalances as measured with HRV, as well as cardiovascular disease, including atrial fibrillation [21], myocardial infarction [22], ischemic stroke [23], and chronic kidney disease [24]. However, reference ranges for non-linear indices of HRV based on Holter ECG recordings had not been reported yet. Sammito and Böckelmann [25, 26] investigated 695 healthy individuals and provided reference values based on 24 h Holter ECG recordings for four time domain and three frequency domain parameters only. In contrast to the present study, participants older than 60 years were excluded [25], which limits generalizability to the population most at risk of cardiovascular disease. Beckers et al. [27] investigated time, frequency, and non-linear parameters in 276 healthy individuals and provided means and standard deviations for various HRV parameters stratified by sex, but did not establish reference values. The present study attempted to fill this gap in the literature for a range of HRV markers, which were selected on the basis of a systematic literature screen as well as a machine learning approach. The former especially resulted in markers from the time and frequency domains, while the latter identified non-linear indices.

The time domain markers, standard deviation of the NN intervals (SDNN) and standard deviation of the 5 min average NN intervals (SDANN), are both markers of overall autonomic function including information from cardiac sympathetic and parasympathetic nervous system activity [6]. However, when derived from Holter records, variations are mostly due to activity of the sympathetic nervous system [28]. The root mean square of the successive differences between normal heart beats (rMSSD) and percentage of neighboring NN intervals that differ from each other by more than 50 ms (pNN50) are parameters mostly reflecting vagal activity from higher frequency oscillations [6].

Ultra-low frequency (ULF) power reflects oscillations in the heart rhythm with a period of ≥ 5 min. Variations in circadian rhythm, internal body temperature, metabolism, and the renin–angiotensin system are all assumed to contribute to differences in ULF spectral power [29–31]. However, the exact contribution of the ANS to ULF power is not clear yet. The physiological mechanisms contributing to variations in the very low frequency (VLF) band are uncertain, but previous experimental research has shown that the intrinsic nervous system of the heart generates the VLF rhythm and efferent sympathetic activity [28]. Traditionally, the HF band in ms/Hz is reported as proxy for parasympathetic tone and the LF band has often been used as proxy for sympathetic activity; more recently, the latter has also been recognized as a proxy of baroreflex sensitivity [32–34]. Combined, the LF/HF ratio is used as a marker of sympathovagal balance.

From the non-linear indices that were identified in this study with machine learning, AC, DC, time lag, embedding dimension, fractal dimension, sample entropy, and the maximal Lyapunov exponent are all measures capturing non-linear, complex heart rhythm dynamics, which are not captured with traditional time and frequency analysis of the RR intervals [35]. The time lag is an intermediate parameter used for the calculation of more complex statistics, and indicates by how much an RR interval series should be shifted with respect to itself until consecutive values are no longer strongly correlated. In sicker individuals, the variability in RR intervals is low (i.e., there is little adaptation of heart rate to dynamic variations in environmental exposures), and so the time between R peaks does not vary much. Hence, a longer time lag is necessary to capture independent (i.e., uncorrelated) signal from RR interval timings in such individuals. The non-linear analysis of HRV is especially important in subjects with cardiovascular disease where HRV is usually depressed, which complicates linear analysis of the RR intervals. With regard to physiological mechanisms of non-linear indices, only AC and DC can separate sympathetic from vagal rhythm modulations of the heart rate [22].

Beyond defining reference values, this investigation has also revealed clinical characteristics that are associated with altered HRV. Besides the well-known age and sex differences in HRV, this study has shown that dyslipidemia and a positive family history of myocardial infarction or ischemic stroke were also strongly related to HRV. Importantly, dyslipidemia was not self-reported, but measured in the current investigation. Dyslipidemia has been associated with impaired endothelial function and increased muscle sympathetic nervous system activity [36]. The inverse relationship between dyslipidemia and rMSSD (root mean square of successive differences between normal heartbeats) and DC in this study may indicate impaired parasympathetic nervous system activity or tone besides sympathetic neural overdrive. Although it is difficult to separate effects of vagal modulations from sympathetic modulations on the heart with HRV analysis, these findings underline the importance of physiological mechanisms that lower the heart rate in individuals with heart failure. The fact that this study shows only weak relationships with several CVRFs and comorbidities after adjustment for a large array of potential confounders, even though these were previously thought to strongly influence HRV, underlines the importance of taking the full clinical profile and medication intake into account when analyzing HRV in individuals with heart failure. Nevertheless, HRV markers without additional information are also promising for risk stratification in practice, as they are competitive with existing risk markers and even contribute additional information.

With regard to heart failure, time domain and non-linear indices seem to be of less importance than the frequency domain. Only LF/HF ratio and ULF wavelet showed a clear relationship with heart failure. Interestingly, of the non-linear indices, only time lag seemed to be of some prognostic importance in heart failure. Whereas time lag is not usually considered a stand-alone marker of autonomic dysfunction, as it is used as a variable for calculation of more complex non-linear markers and is, therefore, usually disregarded in analyses, this study has shown that its prognostic potential may even exceed that of more complex markers.

The strong relationship of HRV values outside the reference range and mortality confirmed the clinical importance of establishing HRV reference ranges. Surprisingly, the commonly used time domain parameters did not have as much prognostic information as the non-linear parameters, underlining the importance of incorporating non-linear HRV indices in cardiovascular research, as was previously suggested by the Task force of the European Society of Cardiology and the North American Society of Pacing and Electrophysiology [37] and the e-Cardiology ESC Working Group and the European Heart Rhythm Association [38].

In terms of clinical outcome, only the LF/HF ratio from the frequency domain and AC, DC and time lag from the non-linear indices of HRV seemed of prognostic importance. This is the first study to examine the relationship between time lag and clinical outcome and show that higher values of time lag, reflecting lower overall HRV, are associated with a worse prognosis. Furthermore, the survival analyses show that increased sympathetic and decreased vagal modulations of the heart rate, respectively, represented by AC and DC, are indicative of a higher risk of death. These results are congruent with previous investigations of AC and DC. Bauer et al. [22] showed that DC is a better predictor of mortality after myocardial infarction than SDNN or AC, especially in patients with an ejection fraction > 30 percent. Similarly, Arsenos et al. [39] found that DC predicted mortality in heart failure patients with severe ventricular dysfunction, and Hayano et al. [40] showed that DC is a predictor of survival after myocardial infarction independent of LVEF. The finding that AC and DC were more convincingly related to outcome in persons with HFpEF than in HFrEF may suggest that individuals with HFpEF suffer more from both sympathetic overdrive and vagal inhibition, which is in line with previous studies [41, 42]. Future studies should elucidate the mechanisms underlying these tentative relationships.

### Strengths and limitations of the study

The use of a data-driven approach alongside a systematic literature-based selection of HRV markers was a strength of this study, allowing the identification of more clinically relevant HRV markers than are usually considered. The unbiased selection of HRV markers in this study has shown that the investigation of autonomic function in heart failure requires more complex markers than those commonly used, such as SDNN, rMSSD, or LF/HF. Moreover, the best studied markers from the literature were less strongly associated with mortality or cardiac death than the top HRV markers from the random survival forest model. No other study to date has established HRV reference ranges for clinically relevant HRV measures from the three domains simultaneously and verified their importance. Another strength of this study was the structured, in-depth clinical phenotyping performed in all participants, which, adjusting for a wide range of potential confounders, allowed rigorous assessment of clinical characteristics affecting HRV measures.

Some limitations of this study should also be noted. First, physiological signals, such as those from Holter ECG recordings, are susceptible to measurement noise and artefacts. Advanced signal processing methods may have the potential to provide a more stable signal beyond what the applied R package ‘RHRV’ [13] and phase-rectified signaling averaging can provide. Second, although this study has shown that associations between HRV and death are undeniably strong, the biological mechanisms underlying these tentative relationships are still unclear. Future studies incorporating molecular data may contribute to a better understanding of the underlying mechanisms. Third, the results have not been validated in another cohort, which should be performed to confirm their generalizability. Moreover, the reference group used in this study was not completely free of (subclinical) disease. However, the cardiovascular status of these individuals, as determined by extensive echocardiography and circulating biomarkers, was within the physiological range and the reference group was free of (the risk of) heart failure. Additionally, sample sizes varied by HF phenotype, hampering the precision of phenotype-specific analyses. Finally, HRV measurements could not be validated as markers of autonomic dysfunction with direct, but invasive measures of the ANS, such as microneurography or muscle sympathetic nerve activity (MSNA). Nevertheless, this study has demonstrated that 24 h ECG-based HRV is a non-invasive modality that allows risk stratification with respect to mortality in both cardiovascular healthy individuals and patients with heart failure. This highlights its clinical relevance and utility in the outpatient setting.

## Conclusion

Holter ECG-based heart rate variability is a non-invasive tool with prognostic relevance in individuals at risk of heart failure and with heart failure. The reference ranges presented in this study may support individualized risk assessment. Regardless of the stage of heart failure, the ability of HRV markers to predict overall survival independent of clinical risk factors, concomitant diseases, and medication could improve risk monitoring in outpatient heart failure management. Future research is required to investigate whether autonomic dysfunction can be specifically targeted to influence disease development.

## Supplementary Information

Below is the link to the electronic supplementary material.

Supplementary file1 (DOCX 5699 kb)

## Data Availability

This project constitutes a major scientific effort with high methodological standards and detailed guidelines for analysis and publication. Data are not made available for the scientific community outside the established and controlled workflows and algorithms. To meet the general idea of verification and reproducibility of scientific findings, we offer access to data at the local database in accordance with the ethics vote on request (contact: Prof. Dr. Philipp Wild (principal investigator of the MyoVasc Study), philipp.wild@unimedizin-mainz.de).
