# Supplementary material for: Heart rate variability: reference values and role for clinical profile and mortality in individuals with heart failure
Source: Clin Res Cardiol. 2023 Jul 9;113(9):1317–30. doi: 10.1007/s00392-023-02248-7 (PMC11371886; doi:10.1007/s00392-023-02248-7)
Supplement: Supplementary file 1 — Supplementary file1 (DOCX 5699 kb) [file 392_2023_2248_MOESM1_ESM.docx]

**SUPPLEMENTAL APPENDIX**

**Heart rate variability: Reference values and role for clinical profile and mortality**

**in individuals with heart failure**

Silav Zeid, MSc^1,2^, Gregor Buch, MSc^1,2,3^, David Velmeden, MD^1,2^, Jakob Söhne, MD^1,2^, Andreas Schulz, PhD^1^, Alexander Schuch, MD^1,2^, Sven-Oliver Troebs, MD^1,2^, Marc William Heidorn, MD^1,2^, Felix Müller, MD^1,2^, Konstantin Strauch, PhD^3^, Katrin Coboeken, PhD^4^, Karl J. Lackner, MD^5,2^, Tommaso Gori, MD^6,2^, Thomas Münzel, MD^6,2^, Jürgen H. Prochaska, MD^1,7,2^, Philipp S. Wild, MD, MSc^1,7,2,8^

^1^Preventive Cardiology and Preventive Medicine, Department of Cardiology, University Medical Center of the Johannes Gutenberg University Mainz, Germany;

^2^German Center for Cardiovascular Research (DZHK), partner site Rhine-Main, Mainz, Germany;

^3^Institute of Medical Biostatistics, Epidemiology and Informatics (IMBEI), University Medical Center of the Johannes Gutenberg University Mainz;

^4^SPM Methods & Applications, Research & Development, Pharmaceuticals; BAYER AG, Wuppertal, Germany;

^5^Institute of Clinical Chemistry and Laboratory Medicine, University Medical Center of the Johannes Gutenberg University Mainz, Germany;

^6^Cardiology I, Department of Cardiology, University Medical Center of the Johannes Gutenberg University Mainz, Germany;

^7^Clinical Epidemiology and Systems Medicine, Center for Thrombosis and Hemostasis (CTH), University Medical Center of the Johannes Gutenberg University Mainz, Germany.

^8^Institute of Molecular Biology (IMB), Mainz, Germany

**Address for correspondence**

Philipp Sebastian Wild, MD, MSc

Professor of Clinical Epidemiology

Preventive Cardiology and Preventive Medicine, Department of Cardiology and

Clinical Epidemiology and Systems Medicine, Center for Thrombosis and Hemostasis

University Medical Center of the Johannes Gutenberg-University Mainz

Langenbeckstr. 1, 55131 Mainz, Germany

Phone: +49 6131 17 7163

Fax: +49 6131 17 8460

Email: philipp.wild@unimedizin-mainz.de

**TABLE OF CONTENT**

Supplemental Methods, Tables and Figures

[**Supplemental Text 1. Extended methods** 3](#_Toc130061092)

[**Supplemental references** 6](#_Toc130061093)

[**Supplemental Table 1.** Overview of all HRV parameters stratified by domain 7](#_Toc130061094)

[**Supplemental Table 2.** Overview on the literature research of HRV parameters ranked by hits 8](#_Toc130061095)

[**Supplemental Figure 1.** Flow chart of sample selection for present analysis 14](#_Toc130061096)

[**Supplemental Table 3.** Heart failure characteristics 15](#_Toc130061097)

[**Supplemental Table 4.** Results from the random survival forest model predicting cardiac death 16](#_Toc130061098)

[**Supplemental Figure 2.** Histograms of the HRV markers in the analysis sample 19](#_Toc130061099)

[**Supplemental Figure 3.** Relationships between clinical profile, and HRV in individuals with heart failure 21](#_Toc130061100)

[**Supplemental Figure 4.** Relationships between clinical profile and HRV with adjustment for CVRFs, comorbidities and medication 23](#_Toc130061101)

[**Supplemental Figure 5A.** Cumulative incidence curves showing the twenty HRV markers in tertiles in relation to all-cause death 25](#_Toc130061102)

[**Supplemental Figure 5B.** Cumulative incidence curves showing the twenty HRV markers in tertiles in relation to cardiac death 29](#_Toc130061103)

[**Supplemental Figure 6A.** Cumulative incidence curves showing inside vs outside of the reference range in relation to all-cause death 33](#_Toc130061104)

[**Supplemental Figure 6B.** Cumulative incidence curves showing inside vs outside of the reference range in relation to cardiac death 37](#_Toc130061105)

[**Supplemental Figure 7.** Relationship of HRV with all-cause death with adjustment for left ventricular ejection fraction 41](#_Toc130061106)

[**Supplemental Figure 8.** Relationship of HRV with all-cause death with adjustment for physical activity 43](#_Toc130061107)

[**Supplemental Figure 9.** Relationship of HRV with cardiac death (A) and outside of the reference range HRV with cardiac death (B) 45](#_Toc130061108)

[**Supplemental Figure 10.** Relationship of HRV with all-cause death in the heart failure phenotypes 47](#_Toc130061109)

**Supplemental Text 1. Extended methods**

**Study participant examination**

Echocardiography was performed on an iE33 echocardiography system using an S5-1 sector array transducer (Philips Healthcare, Hamburg, Germany). Four cardiac cycles were obtained (mean frame rate: 50/s) and for offline analysis transferred into an image archiving system (Xcelera, Philips Healthcare, Hamburg, Germany). Left ventricular ejection fraction (LVEF) was computed using Simpson’s method in the apical four-chamber view. Peak velocity of early (E) diastolic inflow and peak lateral early (E’) diastolic mitral annular velocity were measured during a complete cardiac cycle. All measurements of cardiac structure and function were performed according to the current ASE/EAC recommendations.(1)

Resting heart rate and blood pressure were measured by means of an Omron 705-CP electronic oscillometer in a temperature-regulated room after a rest period of five minutes three times in intervals of three minutes. Afterwards, anthropometric measurements were done, including height, weight, and waist- and hip circumference.

Smokers were dichotomized according to anamnestic data into smoker, including occasional, permanent smokers, and non-smokers, i.e. never and ex-smokers. Active smoking was defined through the computer-assisted personal interview if one of the following criteria was met: smoking one cigarette per day, smoking at least seven cigarettes per week, smoking one package per month, smoking one cigarillo per day, smoking at least seven cigarillos per week or smoking two pipes per day.

Obesity was defined as a body mass index (BMI) > 30kg/m^2^. Type-2 diabetes mellitus was defined by glycated hemoglobin ≥ 6.5%, current intake of antidiabetic drugs, or a diagnosis by a physician. Dyslipidemia was defined by a low-density lipoprotein/high-density lipoprotein ratio > 2.5, appropriate current medication, triglyceride concentration > 150, or if a physician previously diagnosed dyslipidemia.

Arterial hypertension was assumed if anti-hypertensive drugs were taken or mean systolic blood pressure was ≥ 140 mmHg, or if diastolic blood pressure was ≥ 90 mmHg. Positive family history of myocardial infarction or stroke was defined as a first-degree family member with myocardial infarction or stroke at age > 60 if male or > 65 if female. Chronic kidney disease was defined as an estimated glomerular filtration rate [eGFR] < 60 ml/min/1.73m², utilizing the CKD-EPI formula.(2) Information on venous thromboembolism (VTE) and stroke were collected through computer-assisted personal interview or medical records. VTE was defined as ever occurred pulmonary artery embolism and/or deep vein thrombosis. Stroke was defined as a previous history of stroke.

Blood samples were drawn from a cubital vein after a fasting period of at least 5h and subsequently processed for biobanking and measurement of routine blood markers. NT-proBNP was measured via a commercially available Elecsys® 2010 proBNP II immunoassay (Roche Diagnostics, Mannheim, Germany).

**Instructions for participant use of Holter devices**

Study participants were explained how to (re)apply the electrodes and asked to wear the device for twenty-four hours (except during showering/bathing) before returning it to the study center. The participant was instructed (1) to keep at least a 15cm distance between the Holter ECG device and mobile phones, (2) to avoid anti-theft devices in department stores and other high voltage electromagnetic radiation areas, and (3) not to engage in any neuromuscular stimulation activities. The participants were asked to keep a sleep diary to document the wake-up time and bedtime. The same procedure was followed for the 24-hour blood pressure measurement recording device (Cardiomem® SEER 1000, GETEMED, Teltow, Germany).

**Assessment of heart rate variability**

The Holter ECG data was transferred from the device by Bluetooth or USB stick and imported into Holter ECG analysis software (CardioDay® 2.4.3.16, GETEMED) in order to retrieve the RR intervals. Subsequently, The RR intervals were then analyzed and converted to secondary variables with the ‘RHRV’ R package.(3) First the RR intervals were filtered for artifacts and anomalous signals (e.g. ectopic beats) by excluding beats that were too different (±13%) from the previous and following beats or the mean calculated over every 50 beats, and HR <25 bpm or >200 bpm. QRS complexes of individuals with a diagnosis of atrial fibrillation were manually reviewed by a physician for sinus rhythm. For calculation of the time domain parameters, the RR intervals were analyzed in 300-second windows, with the width of bins for the histograms set to 7.8125 milliseconds.

Before computing the frequency domain parameters, the RR intervals were linearly interpolated to ensure equally spaced values at a sampling frequency of 4 Hz. For the frequency analysis of stationary signals, the power spectral density of the RR time series was estimated by the periodogram method using fast Fourier transform. For the frequency analysis of non-stationary signals, the power of the heart rate signal for all four frequency bands were computed for the respective minimum and maximum boundaries of the bands (i.e. power of ULF [0-0.03 Hz], VLF [0.03-0.05 Hz], LF [0.05-0.15 Hz], and HF [0.15-0.4 Hz]), where the size of the spectrogram was set to 300 seconds with a displacement of 10 seconds, using the Short-Time Fourier Transform (STFT). The power of the heart rate signal for all four frequency bands were also computed using the wavelet transform, where the mother wavelet to calculate the spectrogram was set to the Daubechies wavelet ‘d4’. For every non-stationary frequency band, the median and interquartile range (IQR) were computed.

Before the HRV parameters based on non-linear and fractal dynamics were computed, the data was tested for non-linearity, and the phase space reconstruction was solved by using the embedding theorem. Time lag, embedding dimension, correlation dimension, sample entropy and the maximal Lyapunov exponent are all involved in chaotic non-linear analysis with phase space reconstruction. The time lag was estimated by using the average mutual information (AMI) function. The time lags were selected where the AMI function decayed to 1/e of its value at zero. The maximum value was set to 100. The embedding dimension algorithm from Cao (4), using 1,000 points from the time series, was used to estimate the minimum embedding dimension for the RR time series. The correlation sum and dimension were computed for six different embedding dimensions with a radius between one and two hundred. Sample entropy (Kolmogorov-Sinai Entropy) was computed based on the correlation sum, the embedding dimension and the radius of the neighborhood. The maximal Lyapunov exponent represents the divergence rate of close trajectories in a chaotic system and was computed for three different radii: 20, 60 and 100. The extrapolation range was determined for each computation where the range for a linear interpolation was most stable. The dependence between successive RR intervals was computed by dividing two parameters that characterize the Poincaré plot: SD1/SD2. SD1 was computed as the standard deviation of the points perpendicular to the line of identity and SD2 as the standard deviation along the line of identity. The spectral index 1/f noise was computed using the range of frequencies where the regression was performed was between 1e-4 and 1e-2 Hz. For the computation of acceleration capacity and deceleration capacity, we used the same algorithm as Bauer et al. (5), where RR intervals were processed with phase-rectified signal averaging and only non-periodic components are excluded. Compression entropy HRV measures were computed according to Baumert et al. (6) where the RR intervals were binary coded for decreases and increases and then compressed using the LZ77 algorithm.

Missing HRV values were imputed by random forest with the R package “missForest”. Highly skewed variables (i.e. rMSSD, pNN50, total power, VLF [ms/Hz], LF [ms/Hz], HF [ms/Hz], LF/HF [ms/Hz], and sample entropy) were transformed by the natural logarithm to approximate normal distribution before statistical analysis.

# **Supplemental references**

1. Lang RM, Badano LP, Mor-Avi V, Afilalo J, Armstrong A, Ernande L, Flachskampf FA, Foster E, Goldstein SA, Kuznetsova T, Lancellotti P. Recommendations for cardiac chamber quantification by echocardiography in adults: an update from the American Society of Echocardiography and the European Association of Cardiovascular Imaging. *Eur Heart J Cardiovasc Imaging* 2015;**16.3**:233-271.
2. Levey AS, Stevens LA, Schmid CH, Zhang Y, Castro III AF, Feldman HI, Kusek JW, Eggers P, Van Lente F, Greene T, Coresh J. A new equation to estimate glomerular filtration rate. *Ann. Intern. Med* 2009;**150.9**:604-612.
3. Rodriguez-Linares L, Vila XA, Lado MJ, Mendez AJ, Otero A, Garcia CA. RHRV: Heart Rate Variability Analysis of ECG Data. R package version 4.2.6. 2020 https://CRAN.R-project.org/package=RHRV
4. Cao L. Practical method for determining the minimum embedding dimension of a scalar time series. *Phys. D: Nonlinear Phenom.* 1997;**110.1-2**:43-50.
5. Bauer A, Kantelhardt JW, Barthel P, Schneider R, Mäkikallio T, Ulm K, Hnatkova K, Schömig A, Huikuri H, Bunde A, Malik M. Deceleration capacity of heart rate as a predictor of mortality after myocardial infarction: cohort study. *Lancet* 2006;**367.9523**:1674-1681.
6. Baumert M, Baier V, Voss A, Brechtel L, Haueisen J. Estimating the complexity of heart rate fluctuations—an approach based on compression entropy. Fluct. Noise Lett. 2005;**5.04**:L557-L563.

# **Supplemental Table 1.** Overview of all HRV parameters stratified by domain

| **Time domain** | **Frequency domain** | **Non-linear indices** |
| --- | --- | --- |
| Mean HR [1/min] | Total Power [ms/Hz] | 1/f slope |
| Median HR [1/min] | ULF [ms/Hz] | Time lag |
| Skewness RR | VLF [ms/Hz] | Embedding dimension |
| Kurtosis RR | LF [ms/Hz] | Correlation dimension |
| SDNN [ms] | HF [ms/Hz] | Fractal dimension |
| SDANN [ms] | LF/HF | Sample entropy |
| SDNN index [ms] | HF_nu_ [%] | Compression entropy^**^ |
| SDSD [ms] | LF_nu_ [%] | Max. Lyapunov exp. (r=20) |
| pNN50 [%] | ULF median [ms/Hz]^*$^ | Max. Lyapunov exp. (r=60) |
| rMSSD [ms] | VLF median [ms/Hz]^*$^ | Max. Lyapunov exp. (r=100) |
| IRRR [ms] | LF median [ms/Hz]^*$^ | Acceleration capacity |
| MADRR [ms] | HF median [ms/Hz]^*$^ | Deceleration capacity |
| HRVi [ms] | LF/HF median^*$^ | SD1 |
| TINN [ms] | HF_nu_ median [%]^*$^ | SD2 |
|  | LF_nu_ median [%]^*$^ | SD1/SD2 |
|  | ULF IQR [ms/Hz]^*$^ |  |
|  | VLF IQR [ms/Hz] ^*$^ |  |
|  | LF IQR [ms/Hz]^*$^ |  |
|  | HF IQR [ms/Hz]^*$^ |  |
|  | LF/HF IQR^*$^ |  |
|  | HF_nu_ IQR [%]^*$^ |  |
|  | LF_nu_ IQR [%]^*$^ |  |

HR, heart rate; min, minute; ms, millisecond; RR, interval from the onset of one R wave to the onset of the next R wave; SDNN, standard deviation of the NN (normal to normal) intervals in ms; SDANN, standard deviation of the 5-minute average NN intervals; SDNN index, mean of the SDNN for each 5 min segment; SDSD, standard deviation of successive RR interval differences; pNN50%, percentage of neighboring NN intervals that differ from each other by more than 50 ms; rMSSD, root mean square of successive differences between normal heartbeats; IRRR, interquartile range of RR interval; MADRR, median of the absolute differences of RR; HRVi, heart rate variability triangular index; TINN, baseline width of the RR interval histogram; Hz, hertz; ULF, ultra-low frequency; VLF, very low frequency; LF, low frequency; HF, high frequency; HF_nu_, HF in normalized units; LF_nu_, LF in normalized units; IQR, interquartile range; f, frequency; Max, maximal; exp, exponent; r, radius; SD1, Poincaré plot component indicating the width of the fitted ellipse; SD2, Poincaré plot component indicating the length of the fitted ellipse; ^*^, short-time Fourier transform; ^$^, Daubechies wavelet d4 transform; ^**^, compression entropy was computed in 6 different ranges.

# **Supplemental Table 2.** Overview on the literature research of HRV parameters ranked by hits

| **Rank** | **HRV marker** | **Search term HRV marker** | **No. of results for HRV query, and HRV marker** | **No. of results for HRV query, and HRV marker, and cardiovascular** |
| --- | --- | --- | --- | --- |
| 1 | HF [ms/Hz] | ((HF) OR ("High frequency")) | 1,122 | 503 |
| 2 | LF [ms/Hz] | ((LF) OR ("Low frequency")) | 1,044 | 459 |
| 3 | LF/HF | (("LF/HF") OR ("ratio of low and high frequency")) OR ("low to high frequency ratio") | 652 | 294 |
| 4 | SDNN [ms] | (SDNN) | 602 | 253 |
| 5 | rMSSD [ms] | (rMSSD) | 454 | 202 |
| 6 | pNN50 [%] | (pNN50) OR (pNN50%) | 312 | 125 |
| 7 | SDANN [ms] | (SDANN) | 265 | 111 |
| 8 | VLF [ms/Hz] | ((VLF) OR ("Very low frequency")) | 221 | 96 |
| 9 | Total Power [ms/Hz] | (("Total power") OR (TP)) AND (frequency) | 198 | 93 |
| 10 | Mean HR [1/min] | ("mean HR") OR ("mean heart rate") | 180 | 76 |
| 11 | SDSD [ms] | (SDSD) OR ("standard deviation of successive differences") | 159 | 60 |
| 12 | SDNN index [ms] | (("SDNN index") OR (SDNNi)) OR (SDNNindex) | 123 | 54 |
| 13 | HF_nu_ [%] | (("HFnu") OR ("high frequency in normalized units")) OR ("HF in normalized units") | 69 | 35 |
| 14 | LF_nu_ [%] | (("LFnu") OR ("low frequency in normalized units")) OR ("LF in normalized units") | 66 | 32 |
| 15 | ULF [ms/Hz] | ((ULF) OR ("Ultra low frequency")) | 57 | 27 |
| 16 | TINN [ms] | ("Triangular Interpolation of the NN Interval") OR (TINN) OR ("Triangular index") | 54 | 26 |
| 17 | HRVi [ms] | ((HRVi) OR (“HRV index”)) OR ("HRV triangular index") | 47 | 19 |
| 18 | Deceleration capacity | ("Deceleration capacity") | 33 | 17 |
| 19 | SD1/SD2 | ("SD1/SD2") OR ("Poincare plot") | 31 | 14 |
| 20 | Fractal dimension | ("fractal dimension") | 15 | 8 |
| 21 | Acceleration capacity | ("Acceleration capacity") | 12 | 8 |
| 22 | SD1 | ("SD1") | 24 | 7 |
| 23 | SD2 | ("SD2") | 26 | 6 |
| 24 | Sample entropy | ("Sample entropy") OR ("SampEn") | 11 | 6 |
| 25 | Correlation dimension | ("correlation dimension") | 11 | 5 |
| 26 | IRRR [ms] | (IRRR) OR ("interquartile range of RR") | 6 | 4 |
| 27 | 1/f slope | (("1/f slope") OR ("1/f")) | 14 | 4 |
| 28 | Max. Lyapunov exponent | (("Max. Lyapunov exponent") OR ("Lyapunov exponent")) OR ("maximum Lyapunov exponent") | 6 | 4 |
| 29 | Time lag | ("time lag") | 3 | 2 |
| 30 | Skewness RR | ("Skewness R-R") OR ("Skewness RR interval") | 3 | 1 |
| 31 | VLF_median_ (STFT) [ms/Hz] | ("median VLF") OR ("VLF median") OR ("median very low frequency") AND ((Fourier) OR ("Fourier transformation")) | 1 | 1 |
| 32 | Embedding dimension | ("embedding dimension") | 1 | 1 |
| 33 | Compression entropy | (("compression entropy") OR ("entropy compression")) | 1 | 0 |
| 34 | Median HR [1/min] | ("median HR") OR ("median heart rate") | 0 | 0 |
| 35 | Kurtosis RR | ("Kurtosis R-R") OR ("Kurtosis RR interval") | 0 | 0 |
| 36 | MADRR [ms] | (MADRR) OR ("median of the absolute differences between adjacent RR intervals") | 0 | 0 |
| 37 | ULF_median_ (STFT) [ms/Hz] | ("median ULF") OR ("ULF median") OR ("median ultra low frequency") AND ((Fourier) OR ("Fourier transformation")) | 0 | 0 |
| 38 | LF_median_ (STFT) [ms/Hz] | ("median LF") OR ("LF median") OR ("median low frequency") AND ((Fourier) OR ("Fourier transformation")) | 0 | 0 |
| 39 | HF_median_ (STFT) [ms/Hz] | ("median HF") OR ("HF median") OR ("median high frequency") AND ((Fourier) OR ("Fourier transformation")) | 0 | 0 |
| 40 | LF/HF_median_ (STFT) | ((("median LF/HF") OR ("LF/HF median")) AND ((Fourier) OR ("Fourier transformation")) | 0 | 0 |
| 41 | HF_nu median_ (STFT) [%] | ((("median HF in normalized units") OR ("HF in normalized units median")) AND ((Fourier) OR ("Fourier transformation")) | 0 | 0 |
| 42 | LF_nu median_ (STFT) [%] | ((("median LF in normalized units") OR ("LF in normalized units median")) AND ((Fourier) OR ("Fourier transformation")) | 0 | 0 |
| 43 | ULF_IQR_ (STFT) [ms/Hz] | ("IQR ULF") OR ("ULF IQR") OR ("ultra low frequency IQR") AND ((Fourier) OR ("Fourier transformation")) | 0 | 0 |
| 44 | VLF_IQR_ (STFT) [ms/Hz] | ("IQR VLF") OR ("VLF IQR") OR ("very low frequency IQR") AND ((Fourier) OR ("Fourier transformation")) | 0 | 0 |
| 45 | LF_IQR_ (STFT) [ms/Hz] | ("IQR LF") OR ("LF IQR") OR ("low frequency IQR") AND ((Fourier) OR ("Fourier transformation")) | 0 | 0 |
| 46 | HF_IQR_ (STFT) [ms/Hz] | ("IQR HF") OR ("HF IQR") OR ("high frequency IQR") AND ((Fourier) OR ("Fourier transformation")) | 0 | 0 |
| 47 | LF/HF_IQR_ (STFT) | ((("IQR LF/HF") OR ("LF/HF IQR")) AND ((Fourier) OR ("Fourier transformation")) | 0 | 0 |
| 48 | HF_nu IQR_ (STFT) [%] | ((("IQR HF in normalized units") OR ("HF in normalized units IQR")) AND ((Fourier) OR ("Fourier transformation")) | 0 | 0 |
| 49 | LF_nu IQR_ (STFT) [%] | ((("IQR LF in normalized units") OR ("LF in normalized units IQR")) AND ((Fourier) OR ("Fourier transformation")) | 0 | 0 |
| 50 | ULF_median_ (wavelet) [ms/Hz] | ("median ULF") OR ("ULF median") OR ("median ultra low frequency") AND ((wavelet) OR ("wavelet transformation")) | 0 | 0 |
| 51 | VLF_median_ (wavelet) [ms/Hz] | ("median VLF") OR ("VLF median") OR ("median very low frequency") AND ((wavelet) OR ("wavelet transformation")) | 0 | 0 |
| 52 | LF_median_ (wavelet) [ms/Hz] | ("median LF") OR ("LF median") OR ("median low frequency") AND ((wavelet) OR ("wavelet transformation")) | 0 | 0 |
| 53 | HF_median_ (wavelet) [ms/Hz] | ("median HF") OR ("HF median") OR ("median high frequency") AND ((wavelet) OR ("wavelet transformation")) | 0 | 0 |
| 54 | LF/HF_median_ (wavelet) | ((("median LF/HF") OR ("LF/HF median")) AND ((wavelet) OR ("wavelet transformation")) | 0 | 0 |
| 55 | HF_nu median_ (wavelet) [%] | ((("median HF in normalized units") OR ("HF in normalized units median")) AND ((wavelet) OR ("wavelet transformation")) | 0 | 0 |
| 56 | LF_nu median_ (wavelet) [%] | ((("median LF in normalized units") OR ("LF in normalized units median")) AND ((wavelet) OR ("wavelet transformation")) | 0 | 0 |
| 57 | ULF_IQR_ (wavelet) [ms/Hz] | ("IQR ULF") OR ("ULF IQR") OR ("ultra low frequency IQR") AND ((wavelet) OR ("wavelet transformation")) | 0 | 0 |
| 58 | VLF_IQR_ (wavelet) [ms/Hz] | ("IQR VLF") OR ("VLF IQR") OR ("very low frequency IQR") AND ((wavelet) OR ("wavelet transformation")) | 0 | 0 |
| 59 | LF_IQR_ (wavelet) [ms/Hz] | ("IQR LF") OR ("LF IQR") OR ("low frequency IQR") AND ((wavelet) OR ("wavelet transformation")) | 0 | 0 |
| 60 | HF_IQR_ (wavelet) [ms/Hz] | ("IQR HF") OR ("HF IQR") OR ("high frequency IQR") AND ((wavelet) OR ("wavelet transformation")) | 0 | 0 |
| 61 | LF/HF_IQR_ (wavelet) | ((("IQR LF/HF") OR ("LF/HF IQR")) AND ((wavelet) OR ("wavelet transformation")) | 0 | 0 |
| 62 | HF_nu IQR_ (wavelet) [%] | ((("IQR HF in normalized units") OR ("HF in normalized units IQR")) AND ((wavelet) OR ("wavelet transformation")) | 0 | 0 |
| 63 | LF_nu IQR_ (wavelet) [%] | ((("IQR LF in normalized units") OR ("LF in normalized units IQR")) AND ((wavelet) OR ("wavelet transformation")) | 0 | 0 |

PubMed search was performed on the 4^th^ of March 2021. The search results are ranked by frequency in descending order. Search query for HRV was “AND ((((((((((24 hour "heart rate variability") OR (24 h "heart rate variability")) OR (24h HRV))) OR ("24 hour" HRV)) OR ("Twenty-four hour HRV")) OR ("Twenty four hour" "heart rate variability")) OR ("Twenty-four hour" "heart rate variability"))) OR ("Holter ECG"))”. Additional search query for cardiovascular was “AND (cardiovascular)”. Abbreviations, HRV, heart rate variability; STFT, short-time Fourier transform; IQR, interquartile range; HR, heart rate; min, minute; ms, millisecond; RR, interval from the onset of one R wave to the onset of the next R wave; SDNN, standard deviation of the NN (normal to normal) intervals in ms; SDANN, standard deviation of the 5-minute average NN intervals; SDNN index, mean of the SDNN for each 5 min segment; SDSD, standard deviation of successive RR interval differences; pNN50%, percentage of neighboring NN intervals that differ from each other by more than 50 ms; rMSSD, root mean square of successive differences between normal heartbeats; IRRR, interquartile range of RR interval; MADRR, median of the absolute differences of RR; HRVi, heart rate variability triangular index; TINN, baseline width of the RR interval histogram; Hz, hertz; ULF, ultra-low frequency; VLF, very low frequency; LF, low frequency; HF, high frequency; HFnu, HF in normalized units; LFnu, LF in normalized units; IQR, interquartile range; f, frequency; Max, maximal; SD1, Poincaré plot component indicating the width of the fitted ellipse; SD2, Poincaré plot component indicating the length of the fitted ellipse.

**Supplemental Figure 1.** Flow chart of sample selection for present analysis


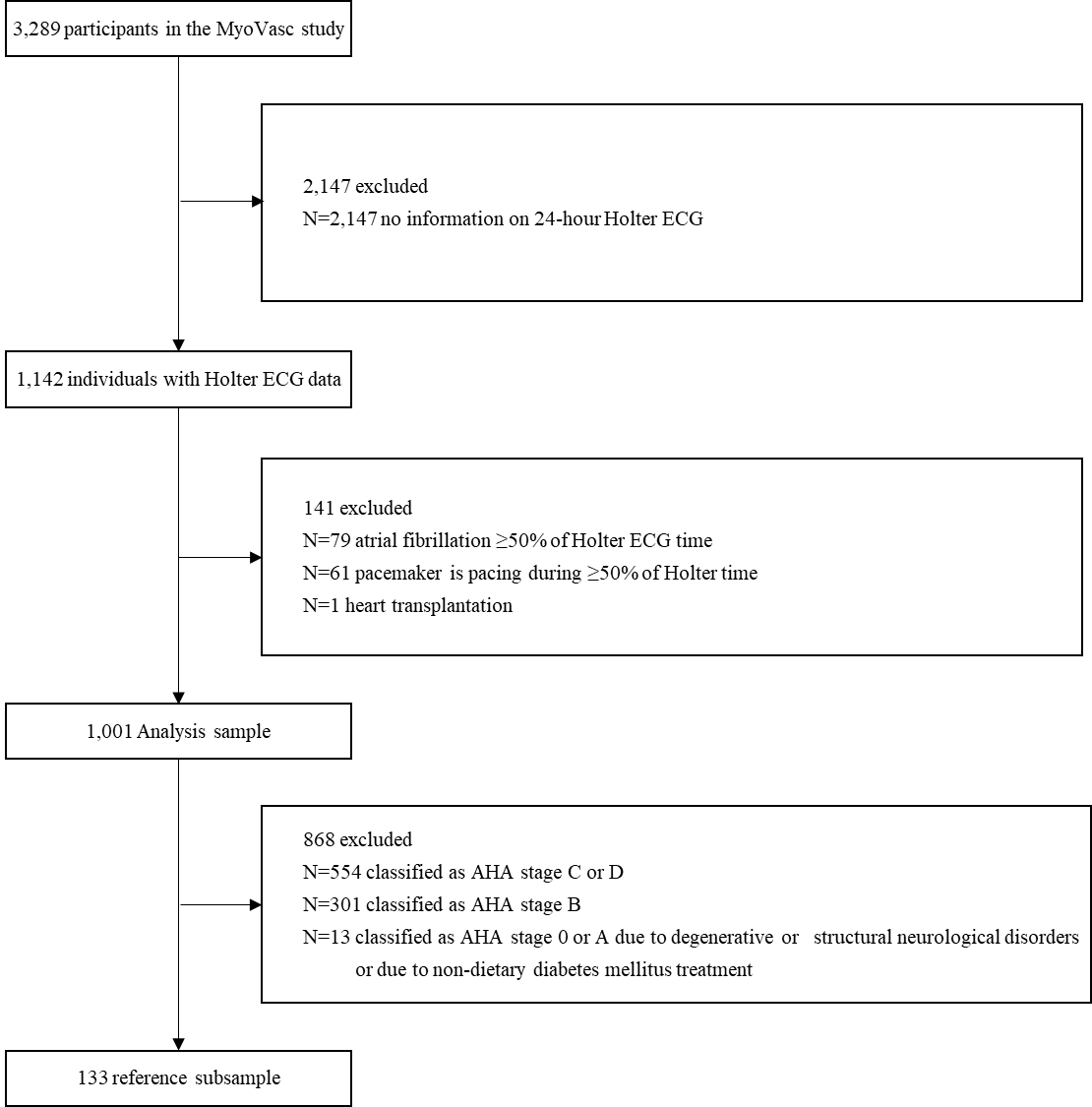


**Supplemental Figure 1:** Flow chart for present analysis, showing the handled exclusions for the subsample selection of the reference group, and individuals with (pre) heart failure.

**Supplemental Table 3.** Heart failure characteristics

|  | Pre-heart failure or heart failure  (n = 855) | Reference group  (n = 133) |
| --- | --- | --- |
| *Heart failure phenotypes* |  |  |
| HFpEF, [%] (n) | 24.0 (205) | 0 (0) |
| HFmrEF, [%] (n) | 13.6 (116) | 0 (0) |
| HFrEF, [%] (n) | 11.6 (99) | 0 (0) |
| *Circulating biomarker* |  |  |
| NT-proBNP, [pg/ml] | 204.1 (96.0/491.2) | 55.5 (38.0/92.1) |
| *Cardiac structure and function* |  |  |
| LVEF, [%] | 53.2 (11.0) | 61.9 (5.1) |
| E/e’ ratio | 9.02 (6.65/11.78) | 6.83 (5.56/8.29) |

Pre heart failure and heart failure are both defined according to the Universal Definition of Heart Failure as stated in the report of the Heart Failure Society of America, Heart Failure Association of the European Society of Cardiology, Japanese Heart Failure Society and Writing Committee of the Universal Definition of Heart Failure, stage B, C or D; The reference group is defined as healthy or at risk of heart failure (HF stage A), excluding individuals with diabetes mellitus diagnosed ≥10 years ago who were not receiving dietary treatment, as well as participants with degenerative or structural neurological disorders. Categorical variables are presented in relative and absolute frequencies. Normally distributed continuous variables are reported as mean (standard deviation), and variables with a skewed distribution as median (quartile 1/quartile 3). HFpEF, heart failure (HF) with preserved ejection fraction (EF); HFmrEF, HF with mildly reduced EF; HF with reduced EF; NT-proBNP, N-terminal prohormone of brain natriuretic peptide; eGFR, estimated glomerular filtration rate; LVEF, left ventricular ejection fraction; E/e’ratio, early mitral inflow velocity divided by mitral annular early diastolic velocity; NYHA, New York Heart Association functional classifications.

# **Supplemental Table 4.** Results from the random survival forest model predicting cardiac death

| **Rank** | **Parameter** | **Minimal depth** |
| --- | --- | --- |
| 1 | Deceleration capacity | 6.100 |
| 2 | Acceleration capacity | 6.622 |
| 3 | Time lag | 6.941 |
| 4 | ULF_median_ (wavelet) [ms/Hz] | 6.949 |
| 5 | Sample entropy | 7.132 |
| 6 | Embedding dimension | 7.228 |
| 7 | Age [SD] | 7.353 |
| 8 | Max. Lyapunov exponent (r=100) | 7.399 |
| 9 | ULF_IQR_ (STFT) [ms/Hz] | 7.427 |
| 10 | Fractal dimension | 7.517 |
| 11 | ULF [ms/Hz] | 7.561 |
| 12 | IRRR [ms] | 7.599 |
| 13 | 1/f slope | 7.600 |
| 14 | HRVi [ms] | 7.656 |
| 15 | Correlation dimension | 7.659 |
| 16 | LF_IQR_ (STFT) [ms/Hz] | 7.723 |
| 17 | ULF_IQR_ (wavelet) [ms/Hz] | 7.785 |
| 18 | LF/HF_IQR_ (STFT) | 7.795 |
| 19 | Max. Lyapunov exponent (r=60) | 7.798 |
| 20 | HF_median_ (STFT) [ms/Hz] | 7.901 |
| 21 | SD1/SD2 | 7.938 |
| 22 | VLF [ms/Hz] | 7.951 |
| 23 | HF_IQR_ (STFT) [ms/Hz] | 7.968 |
| 24 | HF_median_ (wavelet) [ms/Hz] | 7.993 |
| 25 | SDANN [ms] | 8.042 |
| 26 | LF_IQR_ (wavelet) [ms/Hz] | 8.104 |
| 27 | Compression entropy (delta large range) | 8.159 |
| 28 | Total power [ms/Hz] | 8.168 |
| 29 | SDNN [ms] | 8.194 |
| 30 | LF_median_ (STFT) [ms/Hz] | 8.272 |
| 31 | Max. Lyapunov exponent (r=20) | 8.285 |
| 32 | VLF_IQR_ (wavelet) [ms/Hz] | 8.301 |
| 33 | Compression entropy (delta small range) | 8.303 |
| 34 | VLF_IQR_ (STFT) [ms/Hz] | 8.322 |
| 35 | LF_median_ (wavelet) [ms/Hz] | 8.335 |
| 36 | HF_IQR_ (wavelet) [ms/Hz] | 8.335 |
| 37 | HF [ms/Hz] | 8.383 |
| 38 | VLF_median_ (STFT) [ms/Hz] | 8.398 |
| 39 | Kurtosis RR | 8.412 |
| 40 | LF/HF | 8.429 |
| 41 | pNN50 [%] | 8.435 |
| 42 | HF_nu IQR_ (STFT) [%] | 8.457 |
| 43 | SD2 | 8.510 |
| 44 | rMSSD [ms] | 8.526 |
| 45 | HF_nu median_ (STFT) [%] | 8.529 |
| 46 | Skewness RR | 8.541 |
| 47 | LF [ms/Hz] | 8.565 |
| 48 | HF_nu_ [%] | 8.573 |
| 49 | LF/HF_IQR_ (wavelet) | 8.588 |
| 50 | LF/HF_median_ (STFT) | 8.608 |
| 51 | Mean HR [1/min] | 8.636 |
| 52 | Compression entropy (binned delta small range) | 8.660 |
| 53 | HF_nu median_ (wavelet) [%] | 8.669 |
| 54 | Compression entropy (binary, large range) | 8.677 |
| 55 | LF/HF_median_ (wavelet) | 8.721 |
| 56 | ULF_median_ (STFT) [ms/Hz] | 8.724 |
| 57 | SDNN index [ms] | 8.728 |
| 58 | HF_nu IQR_ (wavelet) [%] | 8.733 |
| 59 | VLF_median_ (wavelet) [ms/Hz] | 8.744 |
| 60 | Compression entropy (binned delta large range) | 8.807 |
| 61 | Compression entropy (binary, small range) | 8.913 |
| 62 | Median HR [1/min] | 8.933 |
| 63 | MADRR [ms] | 8.955 |
| 64 | Sex (Women) | 10.747 |

The random survival forest model for competing risks was fitted to rank all HRV markers in relation to cardiac death, including age and sex. The minimum depth, a variable importance metric, was used to rank the markers, where a lower value corresponds to greater importance. The top ten ranked HRV markers were considered clinically important. The minimal depth threshold for selection was 7.671 (N trees = 1000, events = 42, N=1001). Abbreviations: SD, standard deviation; HRV, heart rate variability; STFT, short-time Fourier transform; IQR, interquartile range; HR, heart rate; min, minute; ms, millisecond; RR, interval from the onset of one R wave to the onset of the next R wave; SDNN, standard deviation of the NN (normal to normal) intervals in ms; SDANN, standard deviation of the 5-minute average NN intervals; SDNN index, mean of the SDNN for each 5 min segment; SDSD, standard deviation of successive RR interval differences; pNN50%, percentage of neighboring NN intervals that differ from each other by more than 50 ms; rMSSD, root mean square of successive differences between normal heartbeats; IRRR, interquartile range of RR interval; MADRR, median of the absolute differences of RR; HRVi, heart rate variability triangular index; TINN, baseline width of the RR interval histogram; Hz, hertz; ULF, ultra-low frequency; VLF, very low frequency; LF, low frequency; HF, high frequency; HFnu, HF in normalized units; LFnu, LF in normalized units; IQR, interquartile range; f, frequency; Max, maximal; SD1, Poincaré plot component indicating the width of the fitted ellipse; SD2, Poincaré plot component indicating the length of the fitted ellipse.


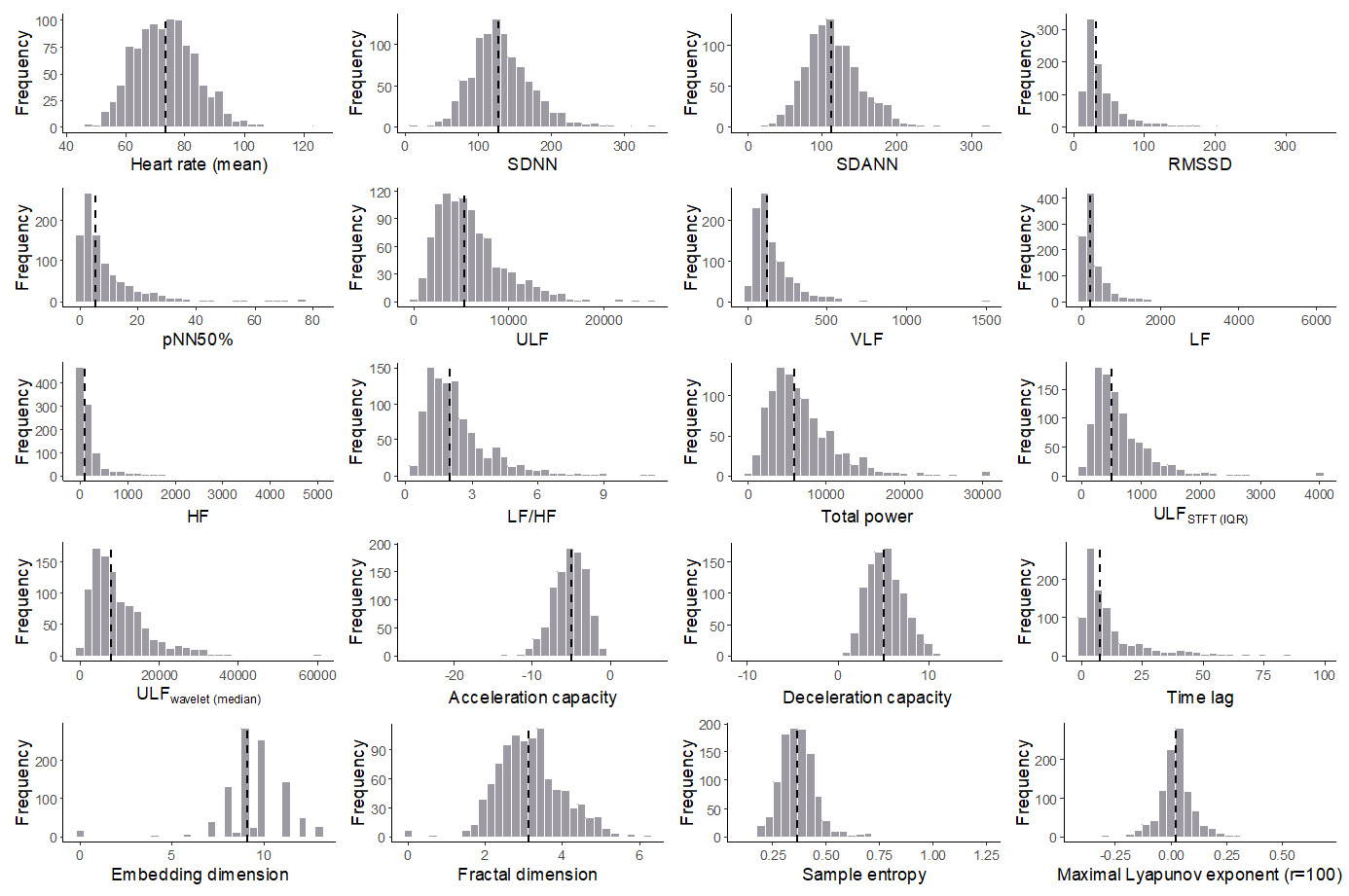
**Supplemental Figure 2.** Histograms of the HRV markers in the analysis sample

**Supplemental Figure 2:** Histograms showing the distribution of the twenty selected heart rate variability markers in the analysis sample (N=1,001); SDNN, standard deviation of the NN (normal to normal) intervals in ms; SDANN, standard deviation of the 5-minute average NN intervals; rMSSD, root mean square of successive differences between normal heartbeats; pNN50%, percentage of neighboring NN intervals that differ from each other by more than 50 ms; ULF, ultra-low frequency; VLF, very low frequency; LF, low frequency; HF, high frequency; STFT, short-time Fourier transform; IQR, interquartile range; r, radius. The median is indicated by the dashed black line.

**Supplemental Figure 3.** Relationships between clinical profile, and HRV in individuals with heart failure

Mean HR

SDNN

SDANN

rMSSD

pNN50

ULF

VLF

LF

HF

LF/HF

Total power

ULF Fourier

ULF wavelet

Acceleration capacity

Deceleration capacity

Time lag

Embedding dimension

Fractal dimension

Sample entropy

Max. Lyapunov exp. (r=100)

Age

Sex

Active smoking

Arterial hypertension

Diabetes mellitus

Dyslipidemia

FHx of MI/stroke

Obesity

Cancer

Chronic kidney disease

Coronary artery disease

Hx of myocardial infarction

Obstructive airway disease/COPD

Peripheral artery disease

Hx of stroke

Symptomatic heart failure

Hx of venous thromboembolism

>0.05

**Negative association**

<0.0005

<0.005

≤0.05

>0.05

<0.0005

<0.005

≤0.05

**Positive association**

**Supplemental Figure 3:** N=855. Each cell shows the coefficient estimate and confidence interval for a cardiovascular risk factor or comorbidity from a separate linear regression model with a HRV marker (standardized) as the dependent variable, adjusted for age and sex. The table is color coded according to the p-values. HR, heart rate; SDNN, standard deviation of the NN intervals; SDANN, standard deviation of the 5-minute average NN intervals; rMSSD, root mean square of the successive differences between normal heart beats; pNN50, percentage of neighboring NN intervals that differ from each other by more than 50 ms; ULF, ultra-low frequency; VLF, very low frequency; LF, low frequency; HF, high frequency; LF/HF, the ratio between low and high frequency; ULF Fourier, interquartile range of the ultra-low frequency short time Fourier transform; ULF wavelet, median of the ultra-low frequency wavelet transform; Max., maximal; r, radius.

# **Supplemental Figure 4.** Relationships between clinical profile and HRV with adjustment for CVRFs, comorbidities and medication

>0.05

**Negative association**

<0.0005

<0.005

≤0.05

>0.05

<0.0005

<0.005

≤0.05

**Positive association**

Mean HR

SDNN

SDANN

rMSSD

pNN50

ULF

VLF

LF

HF

LF/HF

Total power

ULF Fourier

ULF wavelet

Acceleration capacity

Deceleration capacity

Time lag

Embedding dimension

Fractal dimension

Sample entropy

Max. Lyapunov exp. (r=100)

Age

Sex

Active smoking

Arterial hypertension

Diabetes mellitus

Dyslipidemia

FHx of MI/stroke

Obesity

Cancer

Chronic kidney disease

Coronary artery disease

Hx of myocardial infarction

Obstructive airway disease/COPD

Peripheral artery disease

Hx of stroke

Symptomatic heart failure

Hx of venous thromboembolism

**Supplemental Figure 4:** N=855. Each cell shows the coefficient estimate and confidence interval for a cardiovascular risk factor or comorbidity from a separate linear regression model with a HRV marker (standardized) as the dependent variable, adjusted for age and sex, cardiovascular risk factors, comorbidities and medication intake. The table is color coded according to the p-values. HR, heart rate; SDNN, standard deviation of the NN intervals; SDANN, standard deviation of the 5-minute average NN intervals; rMSSD, root mean square of the successive differences between normal heart beats; pNN50, percentage of neighboring NN intervals that differ from each other by more than 50 ms; ULF, ultra-low frequency; VLF, very low frequency; LF, low frequency; HF, high frequency; LF/HF, the ratio between low and high frequency; ULF Fourier, interquartile range of the ultra-low frequency short time Fourier transform; ULF wavelet, median of the ultra-low frequency wavelet transform; Max., maximal; r, radius.

**Supplemental Figure 5A.** Cumulative incidence curves showing the twenty HRV markers in tertiles in relation to all-cause death

**Caption supplemental Figure 5A:** N=855. Panel A. The cumulative incidences of all-cause death in individuals with heart failure over 8 years of follow-up stratified by tertiles are shown for the heart rate variability markers from the time domain. Panel B. The cumulative incidences of all-cause death in individuals with heart failure over 8 years of follow-up stratified by tertiles are shown for the heart rate variability markers from the frequency domain. Abbreviations: Ptot, total power. Panel C. The cumulative incidences of all-cause death in individuals with heart failure over 8 years of follow-up stratified by tertiles are shown for the heart rate variability markers from the non-linear indices.

1.
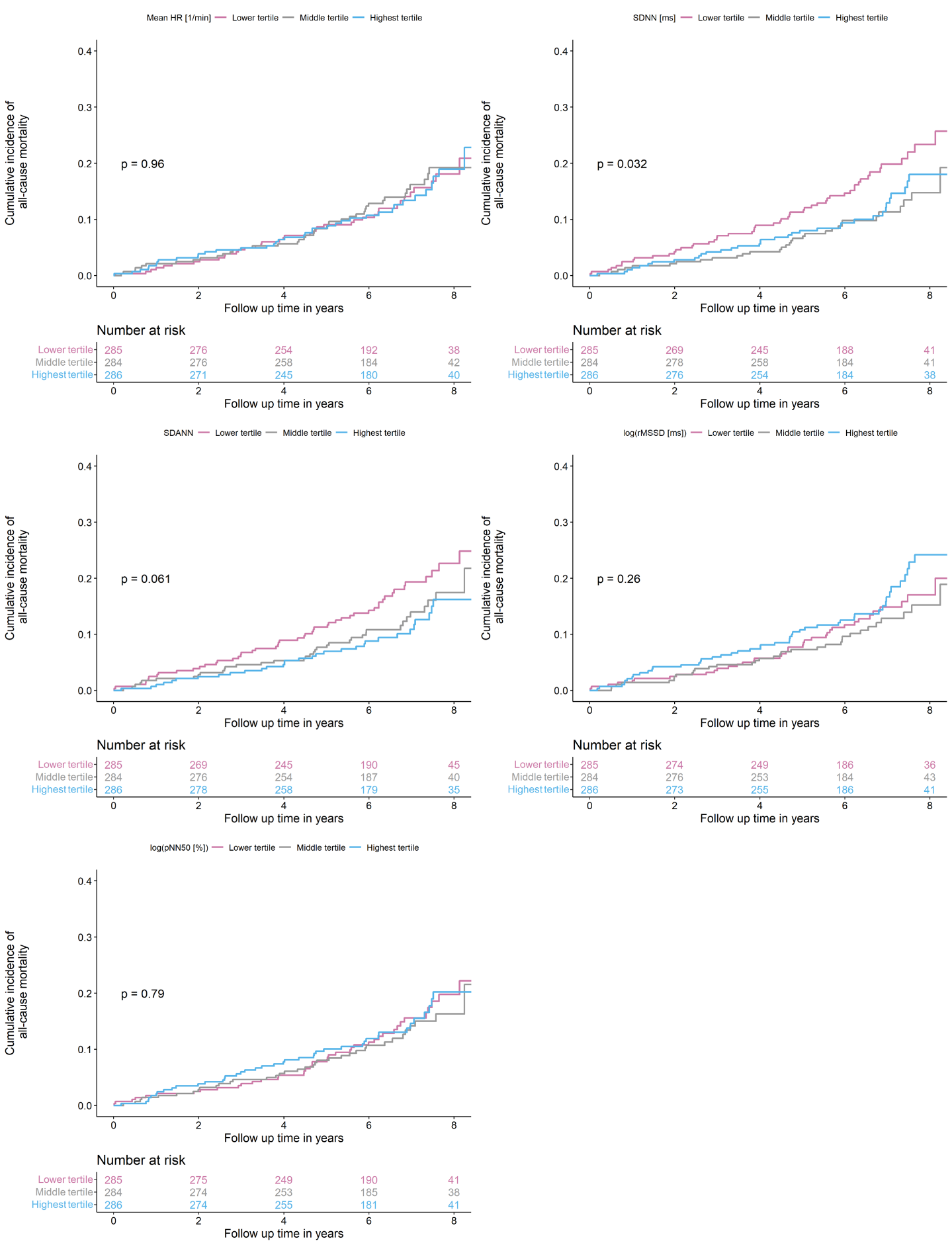
Time domain
2.
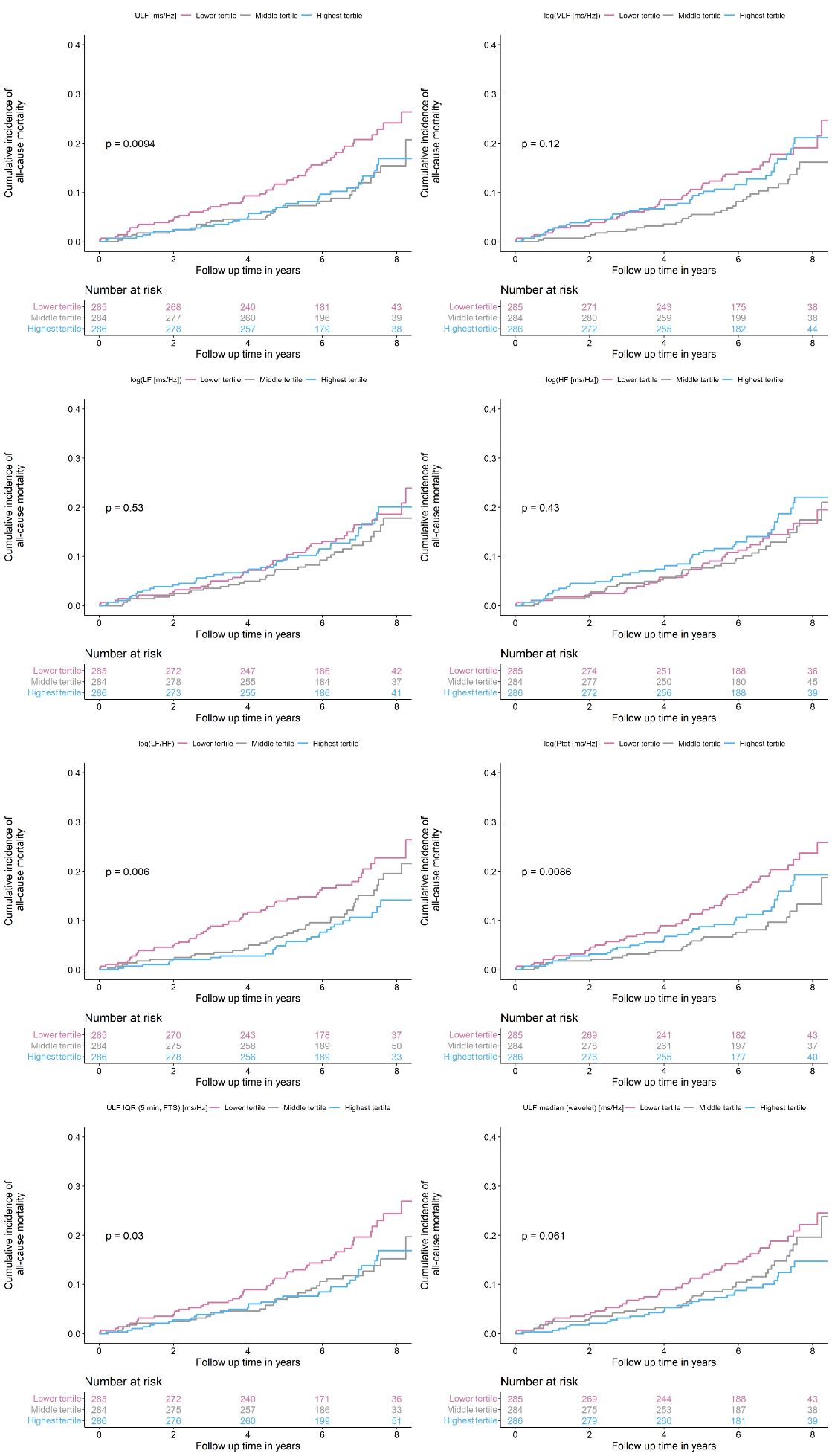
Frequency domain
3.
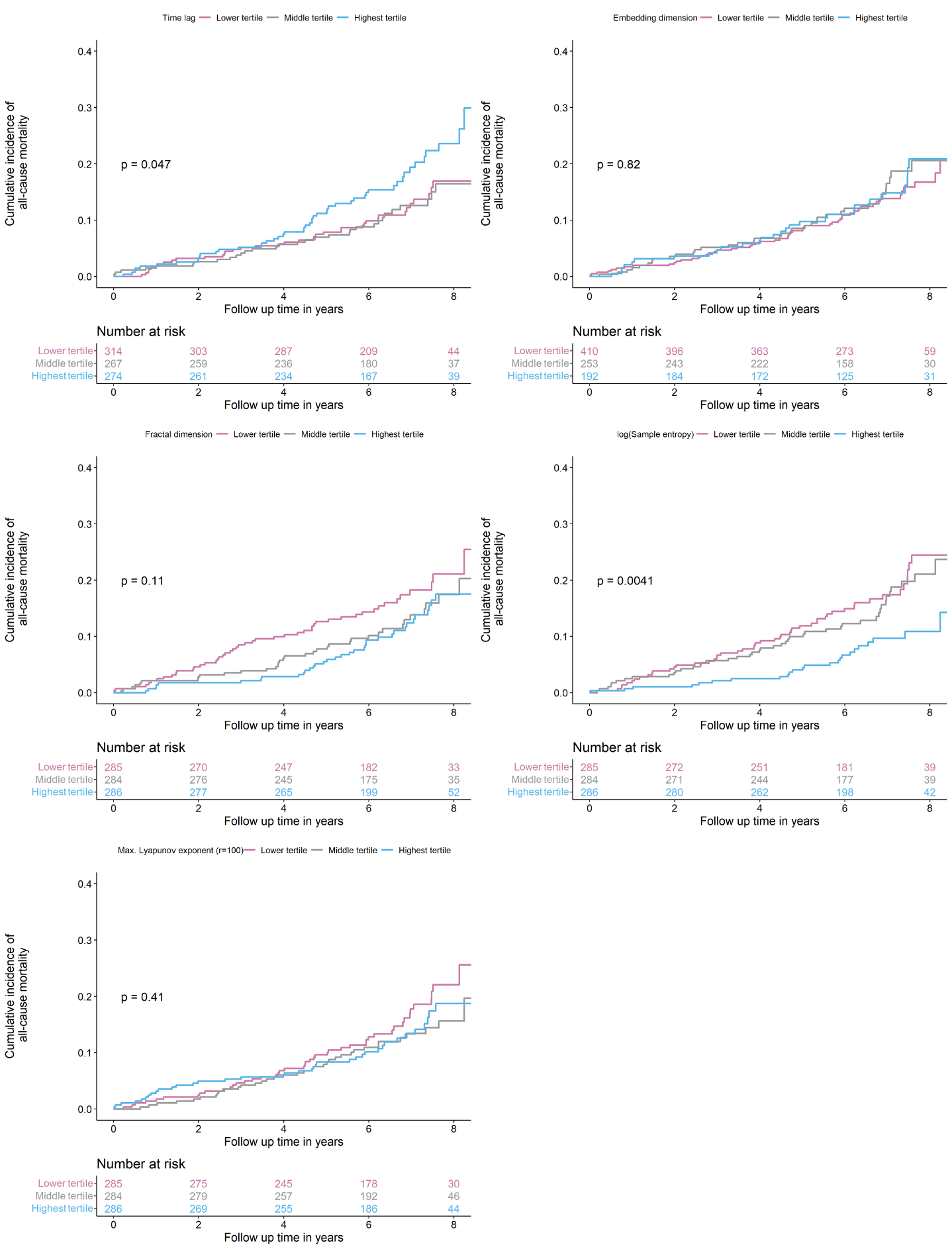
Non-linear indices

**Supplemental Figure 5B.** Cumulative incidence curves showing the twenty HRV markers in tertiles in relation to cardiac death

**Caption supplemental Figure 5B:** N=855. Panel A. The cumulative incidences of cardiac death in individuals with heart failure over 6 years of follow-up stratified by tertiles are shown for the heart rate variability markers from the time domain. Panel B. The cumulative incidences of cardiac death in individuals with heart failure over 6 years of follow-up stratified by tertiles are shown for the heart rate variability markers from the frequency domain. Abbreviations: Ptot, total power. Panel C. The cumulative incidences of cardiac death in individuals with heart failure over 6 years of follow-up stratified by tertiles are shown for the heart rate variability markers from the non-linear indices.

1.
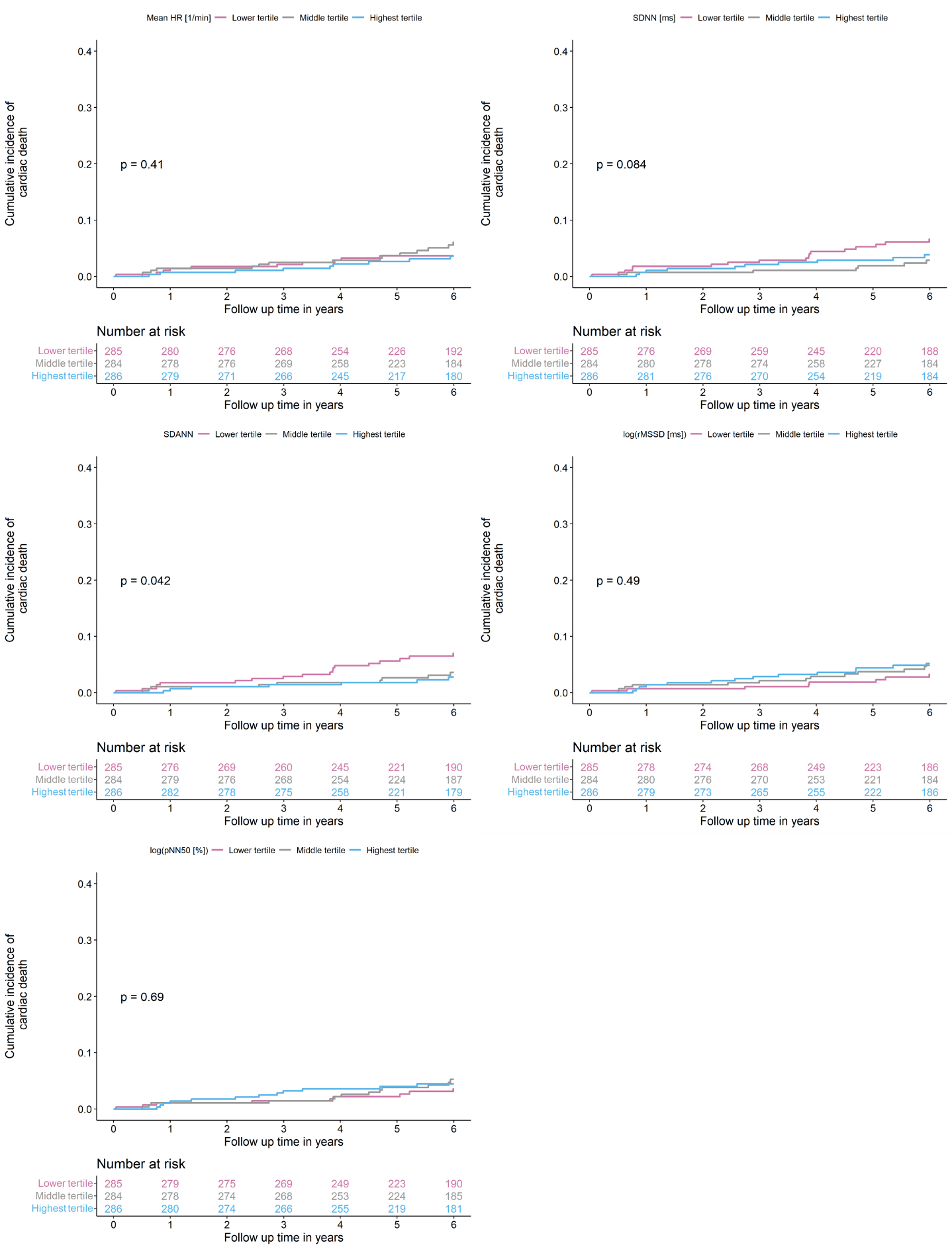
Time domain
2.
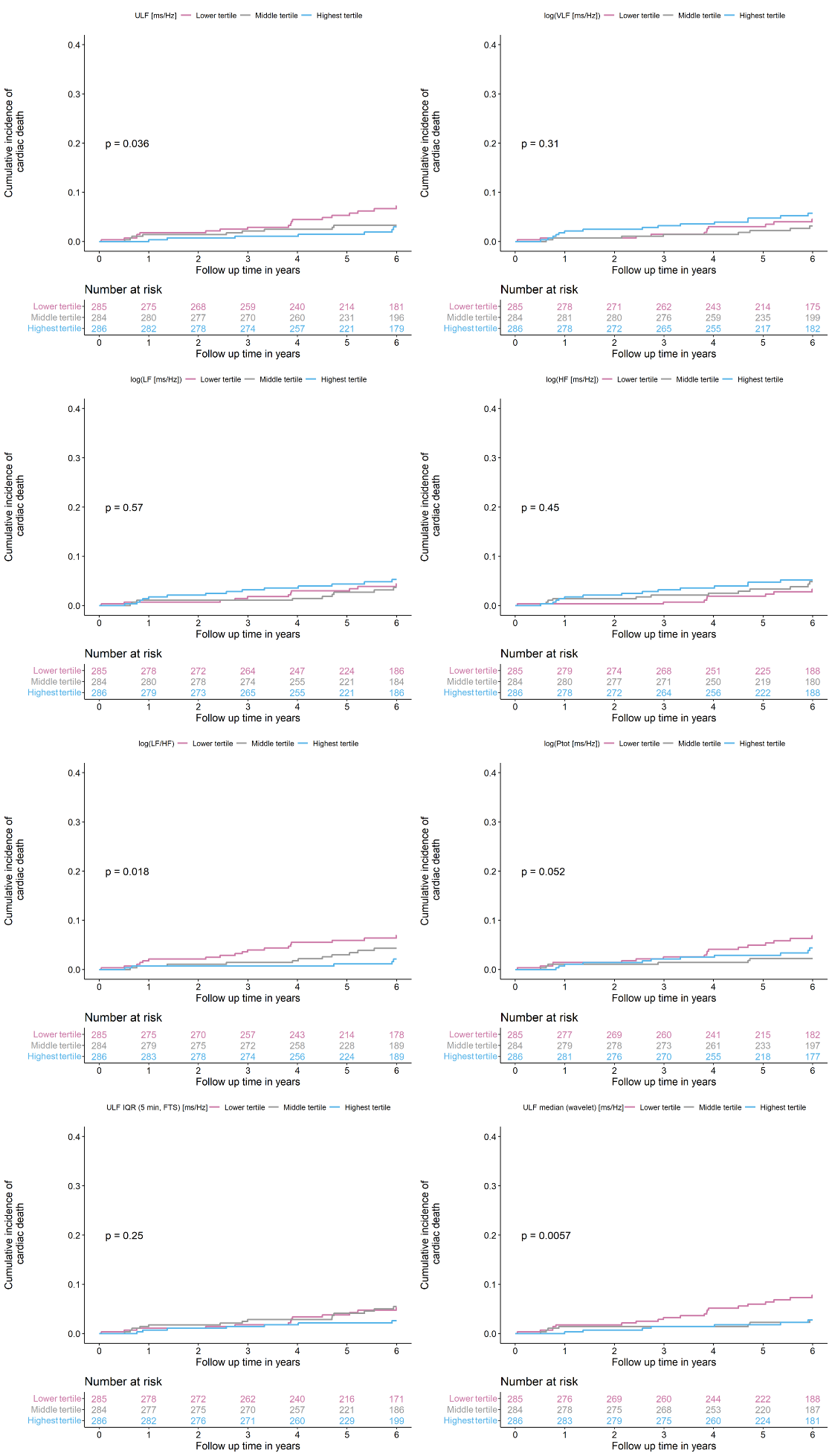
Frequency domain
3.
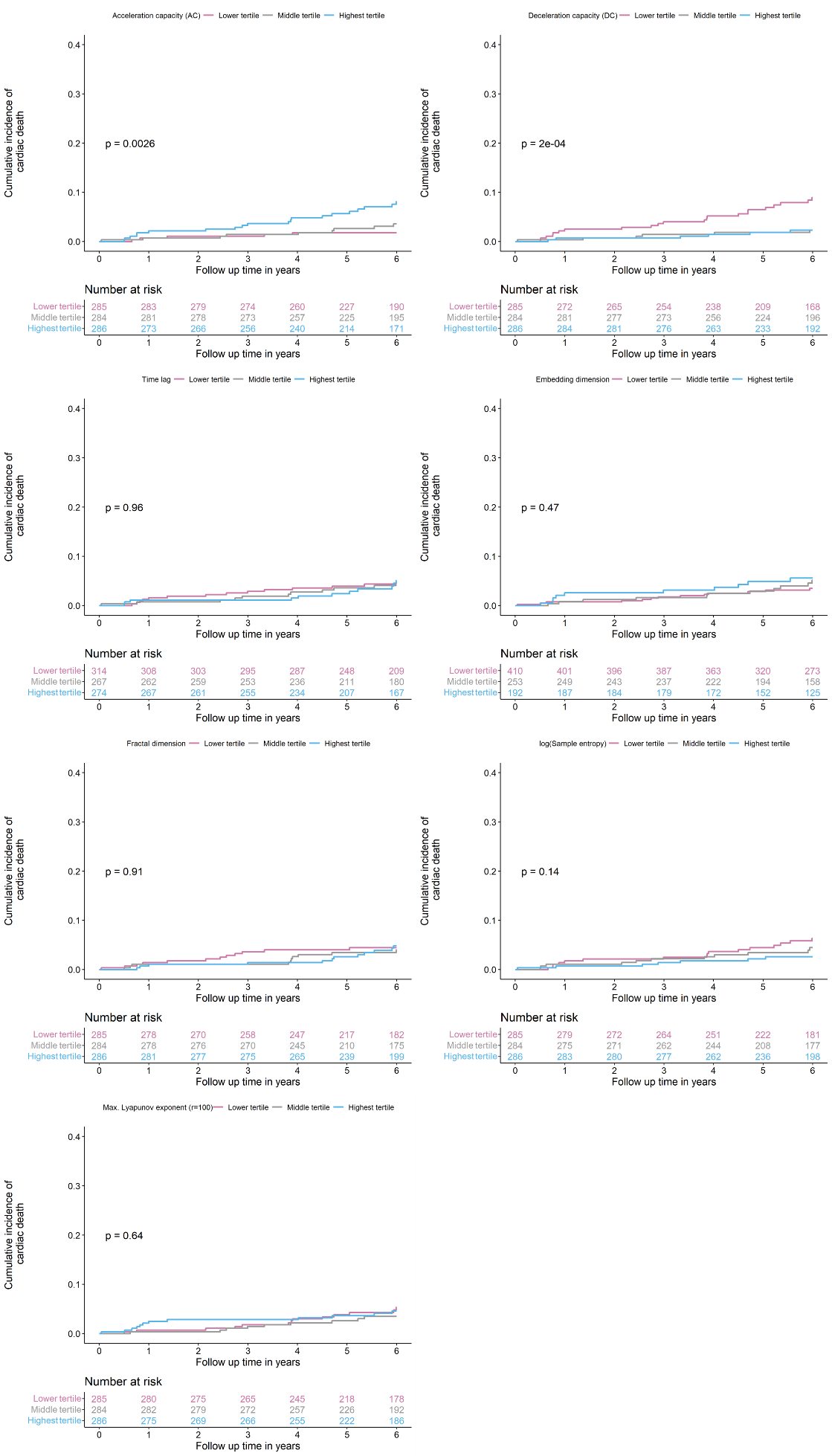
Non-linear indices

**Supplemental Figure 6A.** Cumulative incidence curves showing inside vs outside of the reference range in relation to all-cause death

**Caption supplemental Figure 6A:** N=855. Panel A. The cumulative incidences of all-cause death in individuals with heart failure over 8 years of follow-up stratified by being inside vs outside of the reference range are shown for the heart rate variability markers from the time domain. Panel B. The cumulative incidences of all-cause death in individuals with heart failure over 8 years of follow-up stratified by being inside vs outside of the reference range are shown for the heart rate variability markers from the frequency domain. Abbreviations: Ptot, total power. Panel C. The cumulative incidences of all-cause death in individuals with heart failure over 8 years of follow-up stratified by being inside vs outside of the reference range are shown for the heart rate variability markers from the non-linear indices.

1.
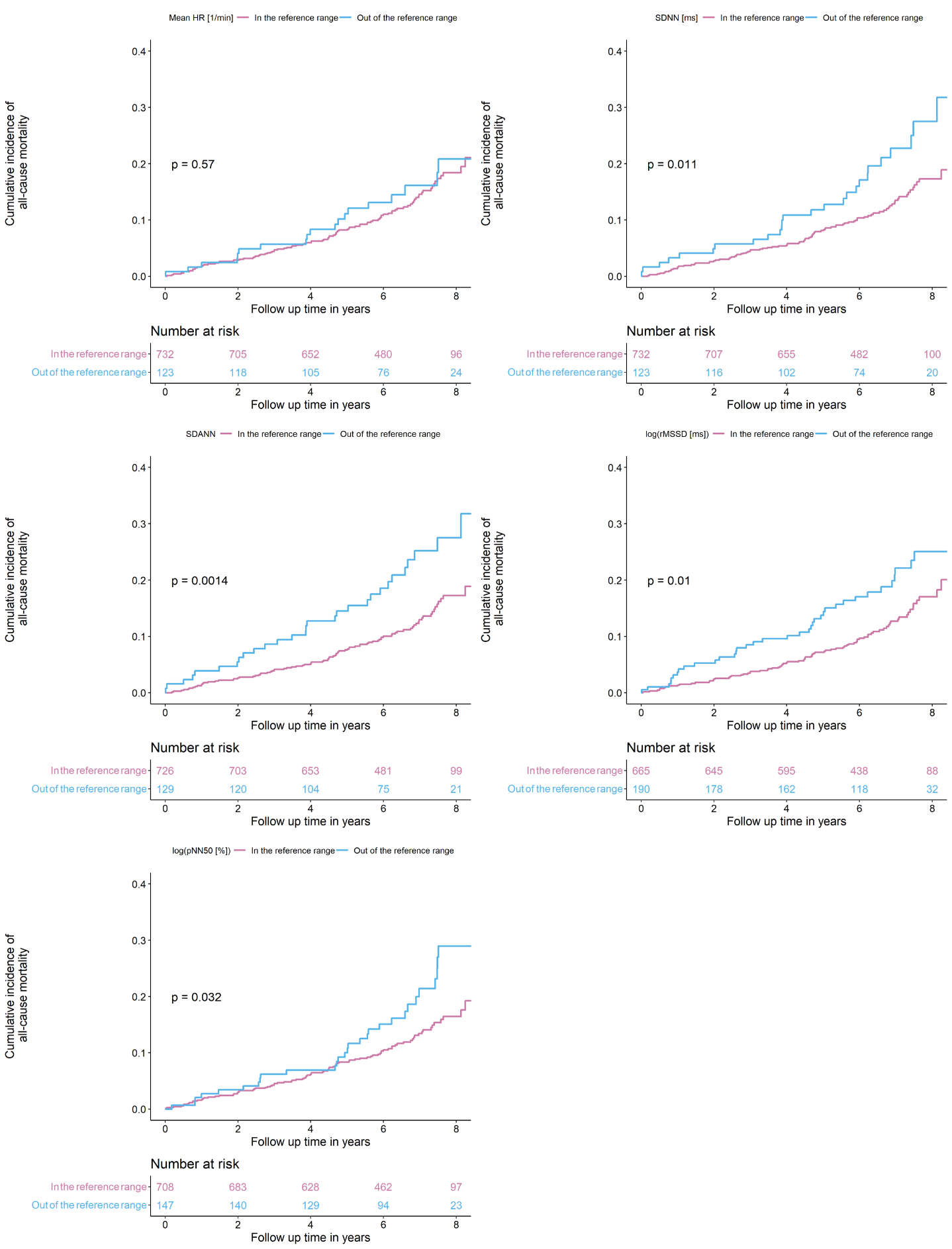
Time domain
2.
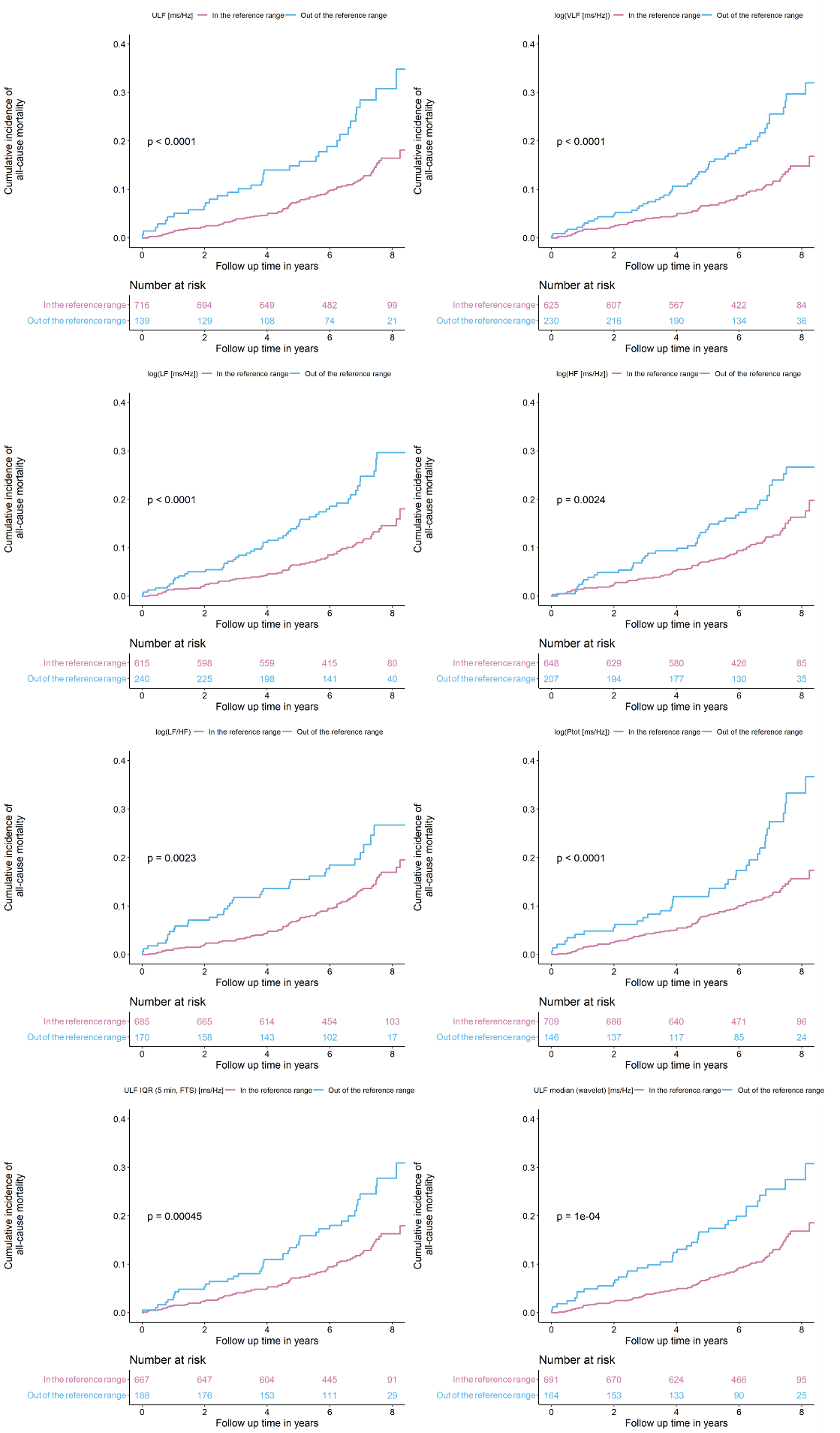
Frequency domain
3.
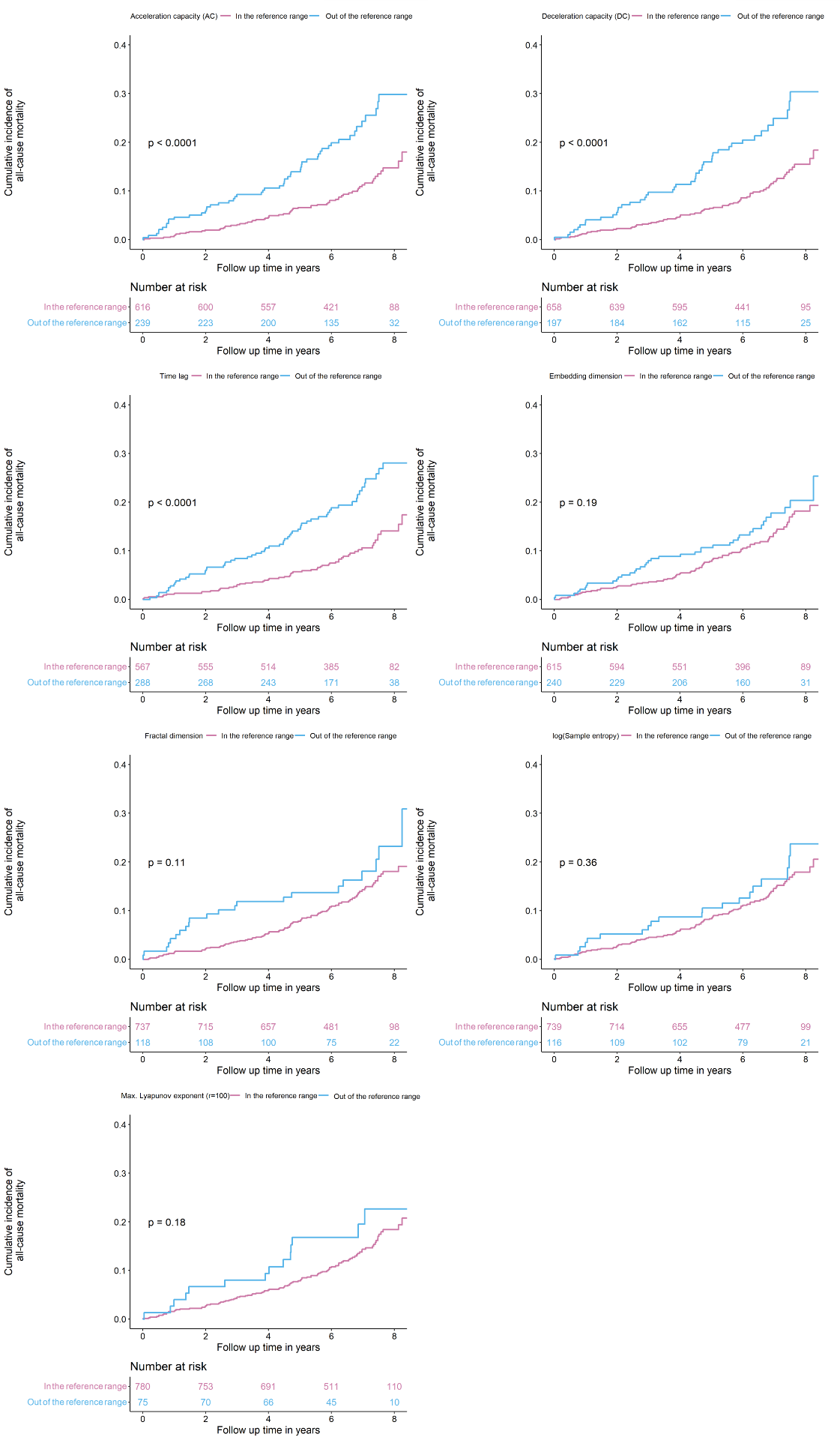
Non-linear indices

**Supplemental Figure 6B.** Cumulative incidence curves showing inside vs outside of the reference range in relation to cardiac death

**Caption supplemental Figure 6B:** N=855. Panel A. The cumulative incidences of cardiac death in individuals with heart failure over 6 years of follow-up stratified by being inside vs outside of the reference range are shown for the heart rate variability markers from the time domain. Panel B. The cumulative incidences of cardiac death in individuals with heart failure over 6 years of follow-up stratified by being inside vs outside of the reference range are shown for the heart rate variability markers from the frequency domain. Abbreviations: Ptot, total power. Panel C. The cumulative incidences of cardiac death in individuals with heart failure over 6 years of follow-up stratified by being inside vs outside of the reference range are shown for the heart rate variability markers from the non-linear indices.

1.
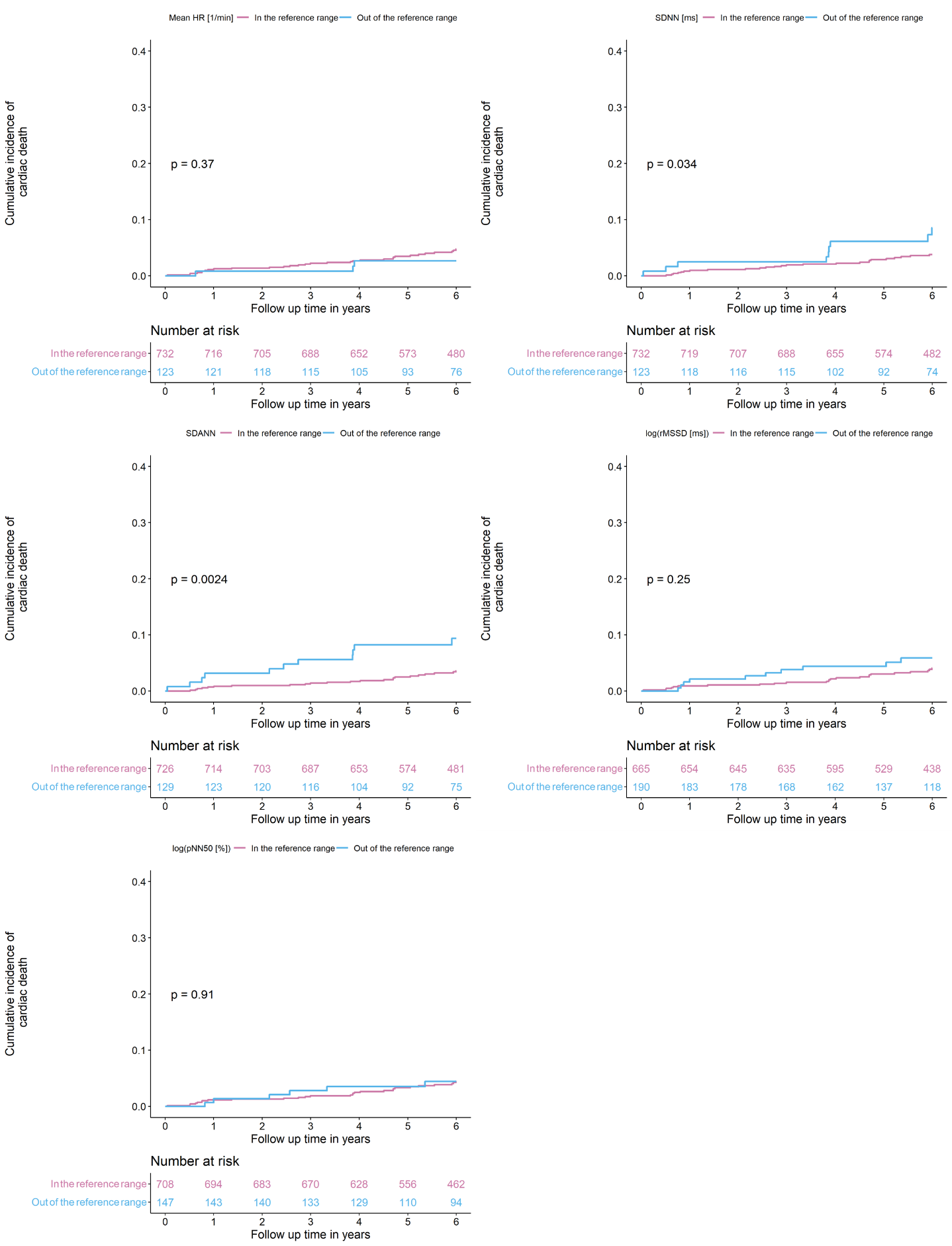
Time domain
2.
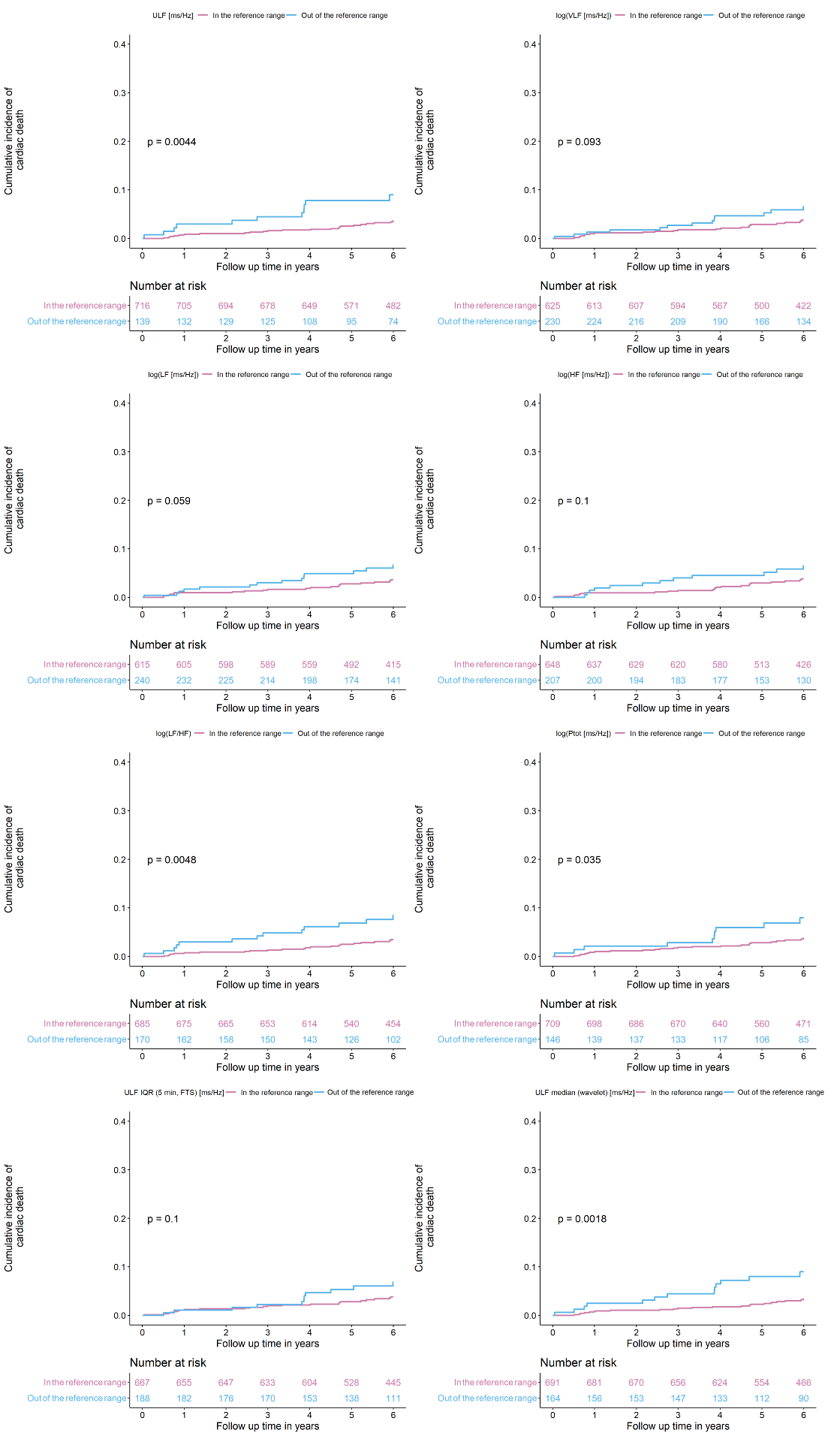
Frequency domain
3.
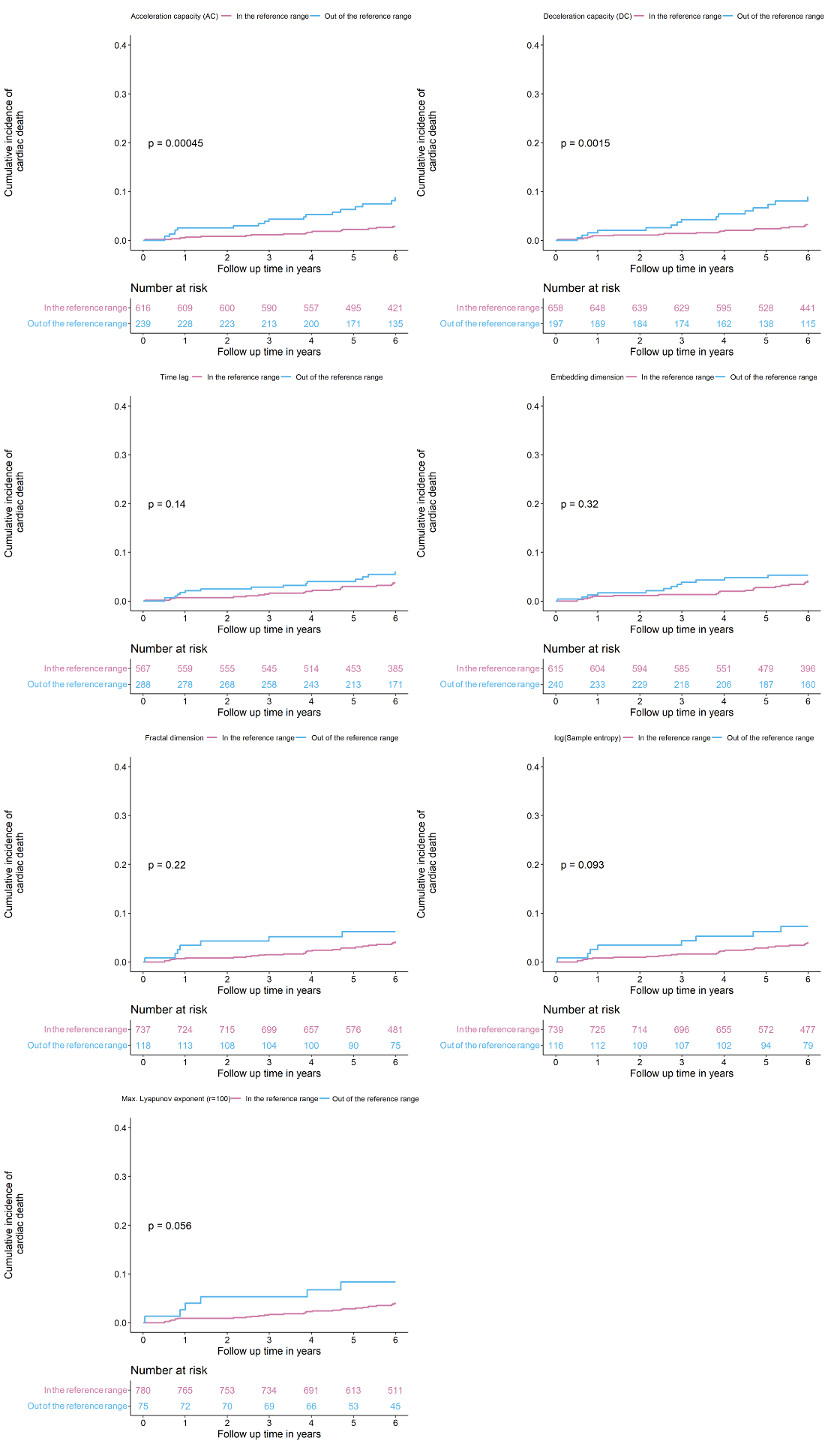
Non-linear indices

**Supplemental Figure 7.** Relationship of HRV with all-cause death with adjustment for left ventricular ejection fraction


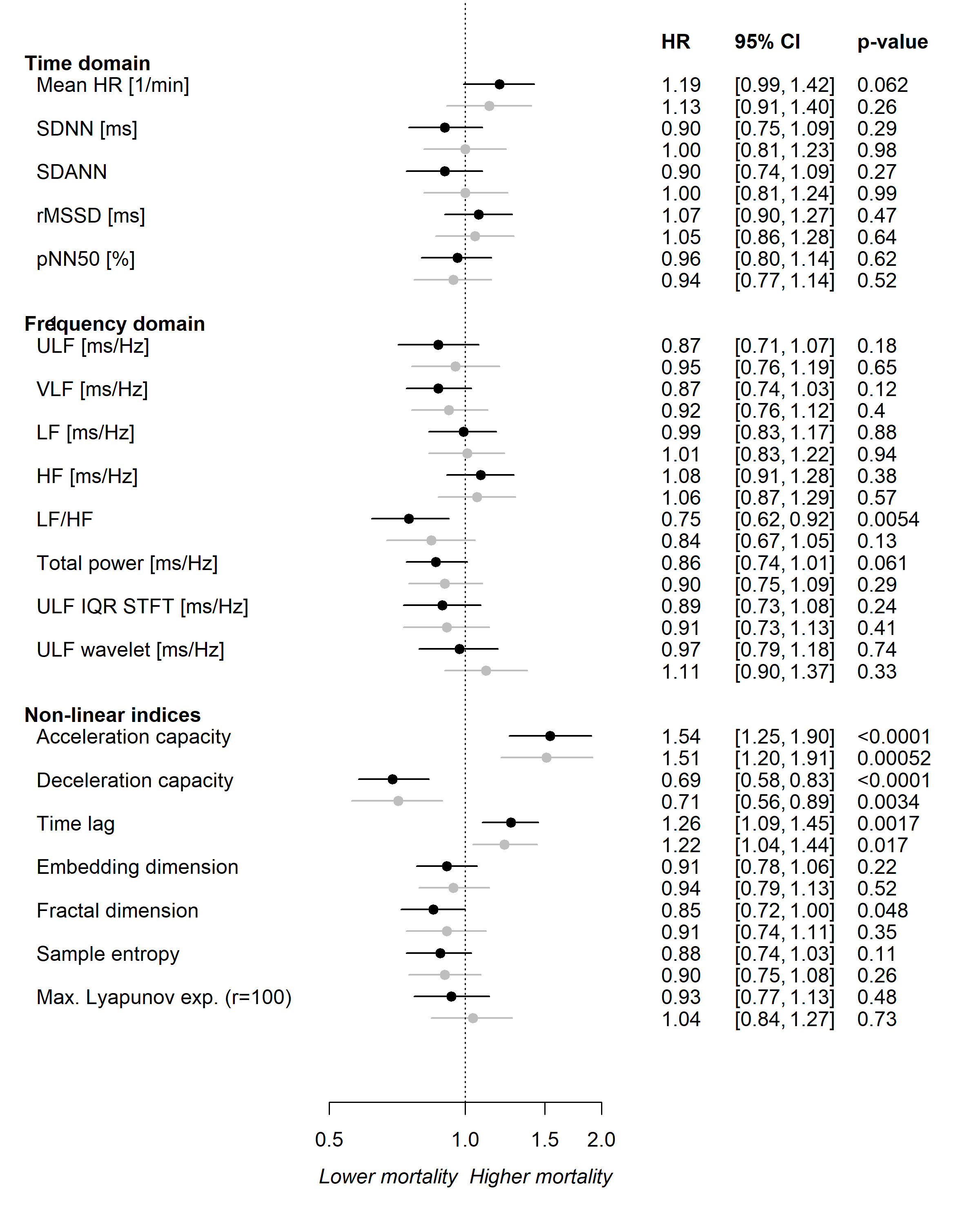


**Supplemental Figure 7:** Results of single Cox regression models, in which the HRV parameter is used as a predictor and all-cause death as the outcome in the (pre) heart failure analysis sample (N=855). In black, the predictor is adjusted for age and sex and left ventricular ejection fraction (LVEF). In grey, additional adjustment for cardiovascular risk factors (i.e. arterial hypertension, diabetes mellitus, smoking, obesity, dyslipidaemia, family history of ischemic stroke or myocardial infarction) and comorbidities (i.e. myocardial infarction, stroke, coronary artery disease, diagnosed peripheral artery disease or ABI <0.9, cancer, diagnosed chronic kidney disease or estimated glomerular filtration rate [eGFR] <60, venous thromboembolism and chronic obstructive pulmonary or airway disease), and medication intake (i.e. antidiabetic agents, antithrombotic agents, cardiac therapy, diuretic agents, beta-receptor blocking agents, selective beta blocking agents, calcium channel blocker, agents acting on the renin-angiotensin system, ACE inhibitors, angiotensin-II-receptor blockers, lipid modifying agents, antidepressants). HR, heart rate; SDNN, standard deviation of the NN intervals; SDANN, standard deviation of the 5-minute average NN intervals; rMSSD, root mean square of the successive differences between normal heart beats; pNN50, percentage of neighbouring NN intervals that differ from each other by more than 50 ms; ULF, ultra-low frequency; VLF, very low frequency; LF, low frequency; HF, high frequency; LF/HF, the ratio between low and high frequency; ULF IQR STFT, interquartile range of the ultra-low frequency short time Fourier transform; ULF wavelet, median of the ultra-low frequency wavelet transform; Max., maximal; r, radius.

**Supplemental Figure 8.** Relationship of HRV with all-cause death with adjustment for physical activity


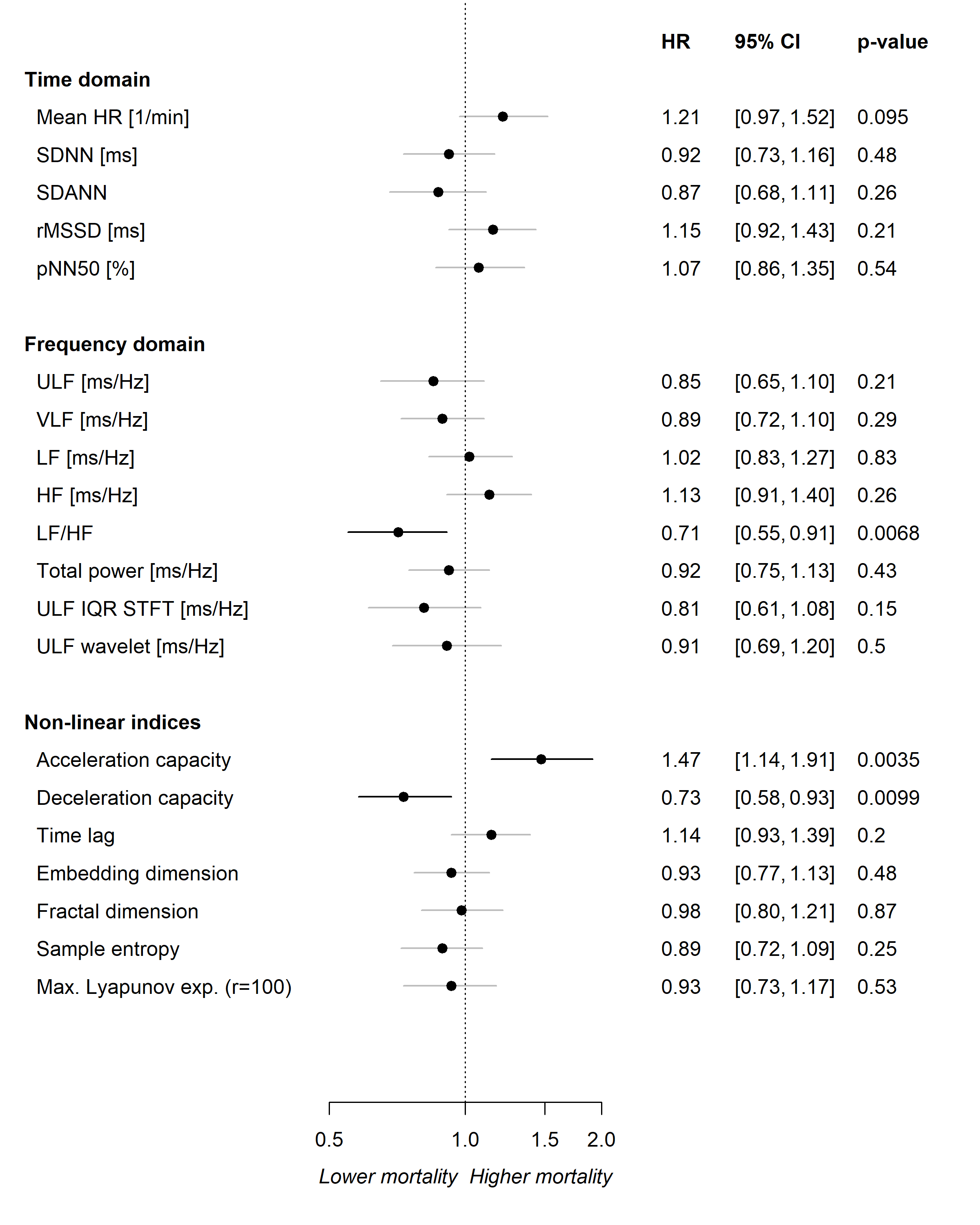


**Supplemental Figure 8:** Results of separate Cox regression models for each HRV parameter in which the HRV parameter is used as a predictor and all-cause death as the outcome with adjustment for age and sex and physical activity (N=458). HR, heart rate; SDNN, standard deviation of the NN intervals; SDANN, standard deviation of the 5-minute average NN intervals; rMSSD, root mean square of the successive differences between normal heart beats; pNN50, percentage of neighboring NN intervals that differ from each other by more than 50 ms; ULF, ultra-low frequency; VLF, very low frequency; LF, low frequency; HF, high frequency; LF/HF, the ratio between low and high frequency; ULF IQR STFT, interquartile range of the ULF short time Fourier transform; ULF wavelet, median of the ULF wavelet transform; Max., maximal; exp., exponent; r, radius.

#
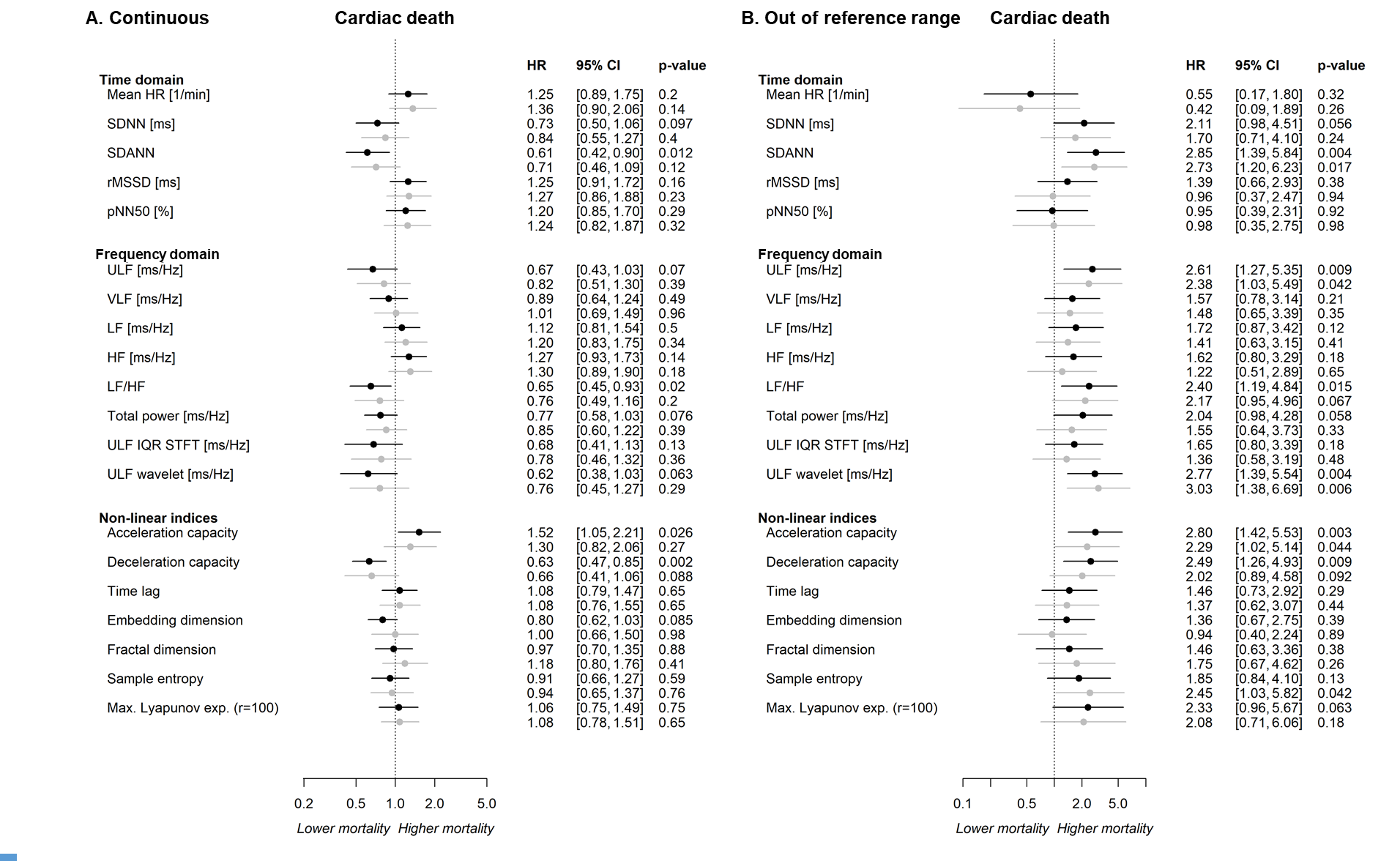
**Supplemental Figure 9.** Relationship of HRV with cardiac death (A) and outside of the reference range HRV with cardiac death (B)

**Supplemental Figure 9:** Panel A. Results of single Cox regression models, in which the HRV parameter is used as a predictor and cardiac death as the outcome in the (pre) heart failure analysis sample (N=855). Panel B. Results of single cox regression models, in which values of being outside vs inside the reference range of the HRV parameter is used as a predictor and cardiac death as the outcome in the (pre) heart failure analysis sample (N=855). In black, the predictor is adjusted for age and sex. In grey, additional adjustment for cardiovascular risk factors (i.e. arterial hypertension, diabetes mellitus, smoking, obesity, dyslipidemia, family history of ischemic stroke or myocardial infarction) and comorbidities (i.e. myocardial infarction, stroke, coronary artery disease, diagnosed peripheral artery disease or ABI <0.9, cancer, diagnosed chronic kidney disease or estimated glomerular filtration rate [eGFR] <60, venous thromboembolism and chronic obstructive pulmonary or airway disease), and medication intake (i.e. antidiabetic agents, antithrombotic agents, cardiac therapy, diuretic agents, beta-receptor blocking agents, selective beta blocking agents, calcium channel blocker, agents acting on the renin-angiotensin system, ACE inhibitors, angiotensin-II-receptor blockers, lipid modifying agents, antidepressants). HR, heart rate; SDNN, standard deviation of the NN intervals; SDANN, standard deviation of the 5-minute average NN intervals; rMSSD, root mean square of the successive differences between normal heart beats; pNN50, percentage of neighbouring NN intervals that differ from each other by more than 50 ms; ULF, ultra-low frequency; VLF, very low frequency; LF, low frequency; HF, high frequency; LF/HF, the ratio between low and high frequency; ULF IQR STFT, interquartile range of the ultra-low frequency short time Fourier transform; ULF wavelet, median of the ultra-low frequency wavelet transform; Max., maximal; r, radius.

**
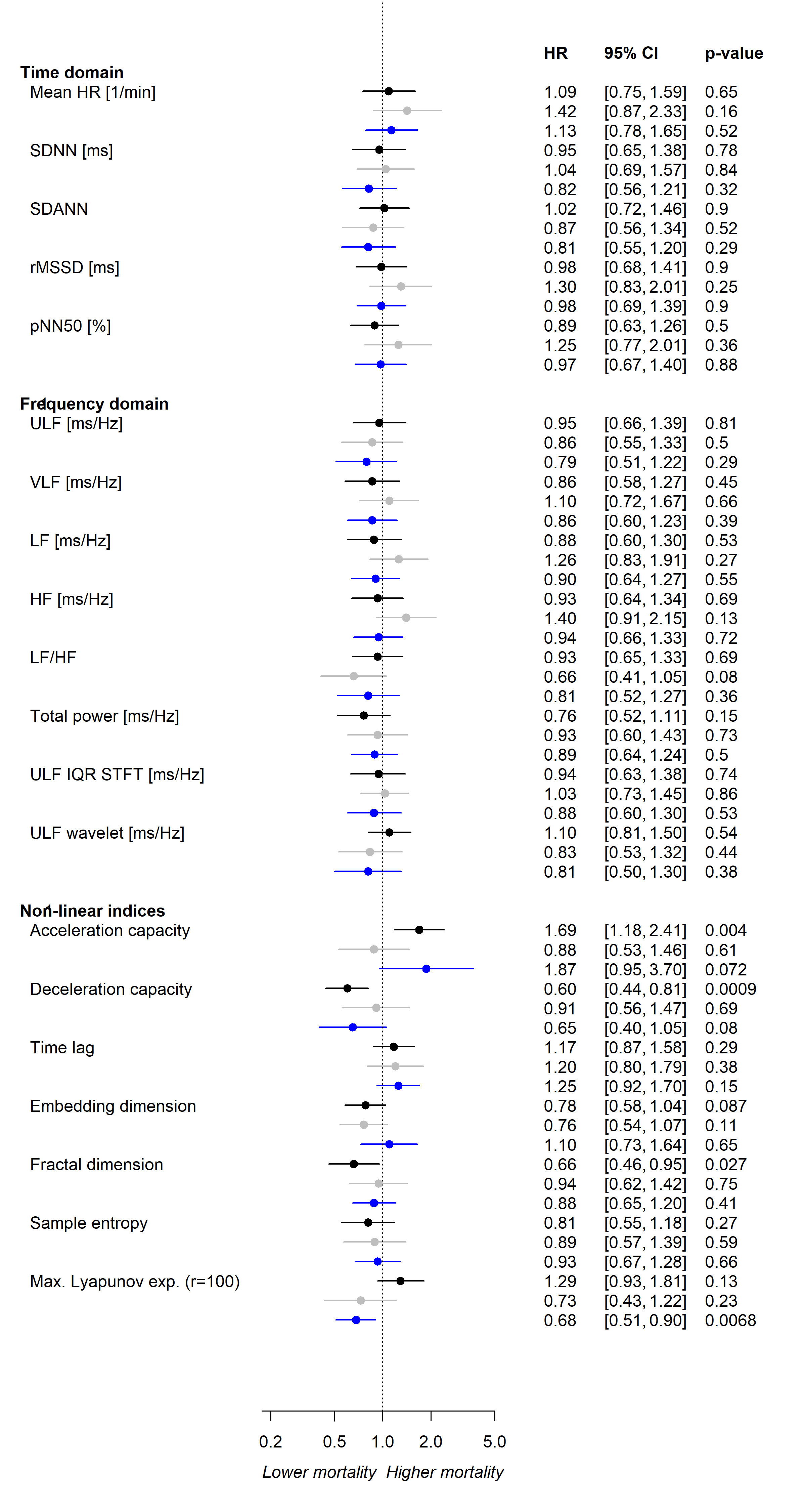
****Supplemental Figure 10.** Relationship of HRV with all-cause death in the heart failure phenotypes

**Supplemental Figure 10:** Results of separate Cox regression models for each HRV parameter in which the HRV parameter is used as a predictor and all-cause death as the outcome with adjustment for age and sex. In black HFpEF (N=205; events=31), in grey HFmrEF (N=116; events=21) and in blue HFrEF (N=99; events=30). HR, heart rate; SDNN, standard deviation of the NN intervals; SDANN, standard deviation of the 5-minute average NN intervals; rMSSD, root mean square of the successive differences between normal heart beats; pNN50, percentage of neighboring NN intervals that differ from each other by more than 50 ms; ULF, ultra-low frequency; VLF, very low frequency; LF, low frequency; HF, high frequency; LF/HF, the ratio between low and high frequency; ULF IQR STFT, interquartile range of the ULF short time Fourier transform; ULF wavelet, median of the ULF wavelet transform; Max., maximal; exp., exponent; r, radius.
